# Therapeutic potential of nicotinamide and ABT263 in alcohol‐associated liver disease through targeting cellular senescence

**DOI:** 10.1002/mco2.70086

**Published:** 2025-02-09

**Authors:** Naheemat Modupeola Gold, Qinchao Ding, Yang Yang, Shaoyan Pu, Wenjing Cao, Xinxuan Ge, Pengyun Yang, Michael Ngozi Okeke, Ayesha Nisar, Yongzhang Pan, Qiuni Luo, Xiayan Wang, Han Xu, Rui Tian, Meiting Zi, Xingjie Zhang, Songtao Li, Yonghan He

**Affiliations:** ^1^ State Key Laboratory of Genetic Evolution & Animal Models Key Laboratory of Healthy Aging Research of Yunnan Province Kunming Institute of Zoology Chinese Academy of Sciences Kunming Yunnan China; ^2^ Kunming College of Life Science University of Chinese Academy of Sciences Kunming China; ^3^ Department of Nutrition and Food Hygiene, School of Public Health Zhejiang Chinese Medical University Hangzhou Zhejiang China; ^4^ Department of Medicine Massachusetts General Hospital Harvard Medical School Boston Massachusetts USA; ^5^ Biodiversity Data Center of Kunming Institute of Zoology Chinese Academy of Sciences Kunming Yunnan China; ^6^ Guangdong Key Laboratory of Nanomedicine Institute of Biomedicine and Biotechnology Shenzhen Institute of Advanced Technology Chinese Academy of Sciences Shenzhen China; ^7^ Department of Ultrasonography The First Affiliated Hospital of Kunming Medical University Kunming Yunnan China; ^8^ Key Laboratory of Medicinal Chemistry for Natural Resource Ministry of Education Yunnan Characteristic Plant Extraction Laboratory Yunnan Key Laboratory of Research and Development for Natural Products State Key Laboratory for Conservation and Utilization of Bio‐Resources in Yunnan School of Pharmacy and School of Chemical Science and Technology Yunnan University Kunming Yunnan China

**Keywords:** acetaldehyde, alcohol‐associated liver disease, cellular senescence, senolytic, senomorphic

## Abstract

Alcohol‐associated liver disease (ALD) is a major cause of liver‐related morbidity and mortality, yet clinically effective therapies for ALD remain lacking. Here, we demonstrate that alcohol intake and its metabolite, acetaldehyde (ACH), induce senescence in the liver and liver cells, respectively. To assess the therapeutic potential of targeting liver senescence in ALD, we treated ALD‐affected mice with the senolytic compound ABT263 and the senomorphic NAD^+^ precursor, nicotinamide (NAM). The results show that ABT263 effectively clears senescent hepatocytes and stellate cells, and reduces liver triglyceride (TG), but increases plasma alanine aminotransferase and TG levels. Conversely, NAM efficiently suppresses senescence and the senescence‐associated secretory phenotype (SASP), protecting the liver from alcohol‐induced injury in ALD mice. RNA‐sequencing analysis revealed that ABT263 treatment downregulated genes involved in adipogenesis while activating the complement pathway. In contrast, NAM upregulated metabolism‐related genes, such as *Sirt1*, and downregulated DNA damage marker genes, including *Rec8* and *E2f1*, in the liver. These findings suggest that cellular senescence plays a critical role in alcohol‐induced liver injury. Compared with senescent cell clearance by ABT263, suppressing senescence and SASP by NAM may provide a safer and more effective therapeutic approach for ALD.

## Graphical abstract

Alcohol metabolite acetaldehyde (ACH) induces senescence in the liver, which is involved in alcohol‐associated liver disease (ALD). Clearance of senescent liver cells with ABT263 reduced triglyceride (TG) in the liver, but increased ALT levels possibly via activation of the complement system. In contrast, suppression of senescence with nicotinamide (NAM) mitigates ALD via inhibiting inflammation and improving metabolism in the liver.

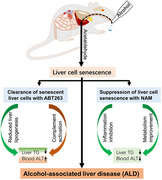

## 1 INTRODUCTION

Alcohol‐associated liver disease (ALD) is a highly prevalent liver disease worldwide, commonly caused by excessive and prolonged alcohol consumption. It encompasses a spectrum of liver disorders, ranging from alcohol‐related steatosis to cirrhosis. Approximately 20–40% of patients with hepatic steatosis due to ALD progress to steatohepatitis, which can further develop into hepatic fibrosis, cirrhosis, and even hepatocellular carcinoma.^1^ Over the last decade, hospitalizations and the overall disease burden related to ALD have increased markedly, resulting in higher healthcare utilization, rising costs, and escalating rates of morbidity and mortality.^2^

Abstinence from alcohol is the most critical factor influencing outcomes at nearly all stages of ALD.^3^ However, sustaining abstinence is challenging for many individuals, despite the known health risks. Currently, corticosteroids (such as prednisolone) are recommended as the first‐line treatment for severe ALD, but their efficacy remains limited.^4^, ^5^ Orthotopic liver transplantation is an option for patients with decompensated ALD cirrhosis or severe alcohol‐related hepatitis. Although early liver transplantation can improve survival in ALD patients,^6^ only a minority achieve recompensated liver function.^7^ The high cost and limited availability of donor organs further constrain liver transplantation as a viable treatment option for advanced ALD. Given the limitations of current treatment options, there is an urgent need for novel therapeutic approaches to treat ALD effectively.

Understanding the mechanisms by which alcohol leads to ALD is essential for developing new therapies. The pathophysiology of ALD is complex, with alcohol‐induced oxidative stress playing a major role in liver injury.^8^ Oxidative stress often results in cellular senescence characterized by irreversible cell cycle arrest. Other characteristics of cellular senescence include the upregulation of cell cycle inhibitors like p21 and p16, activation of antiapoptotic pathways, senescence‐associated secretory phenotype (SASP), and other phenotypic changes.^9^ Cellular senescence can be physiological or pathological depending on the specific situation.^10^ For example, cellular senescence is an essential cell‐autonomous tumor‐suppressor mechanism characterized by an irreversible growth arrest to prevent the proliferation of damaged cells.^11^ However, senescent cells (SnCs) can also promote tumor growth by secreting SASP factors.^12^ Emerging evidence suggests that cellular senescence contributes to the pathogenesis and progress of nonalcohol‐related fatty liver disease (NAFLD),^13^ and targeting senescent hepatocytes can ameliorate NAFLD progression.^14^, ^15^ In ALD patients, the senescence marker p21 was found to be increased and associated with fibrosis stage and adverse liver‐related outcomes.^16^ However, due to the differences in pathogenic factors, progress, duration, and so on, between NAFLD and ALD, plus the high heterogeneity and dual roles of SnCs, it is still unclear whether long‐term alcohol intake is sufficient to induce senescence in the liver and whether targeting senescence improves or aggravates liver function in ALD. Since 2015, small molecules targeting proteins within SnC antiapoptotic pathways have been identified as senolytics, selectively eliminating SnCs.^17^, ^18^, ^19^ Conversely, agents that can suppress senescence or SASP production are referred to as senomorphics. Together, senolytics and senomorphics (collectively termed senotherapeutics) show potential for preventing and treating age‐related diseases and extending healthspan.^20^

In this study, we found that short‐term exposure to alcohol metabolite acetaldehyde (ACH) is sufficient to induce senescence in liver cells. Based on this finding, we investigated whether targeting senescence through either SnC clearance or suppression could serve as a therapeutic approach for ALD. We used ABT263 (navitoclax), a potent senolytic targeting antiapoptotic proteins Bcl‐xL and Bcl‐2,^17^, ^21^, ^22^ which has been previously tested in patients with hepatocellular carcinoma^23^ and in mice with acute‐on‐chronic liver failure induced by lipopolysaccharide and carbon tetrachloride.^24^ In addition, we examined nicotinamide (NAM), a NAD^+^ precursor known to reduce oxidative stress and commonly studied in NAFLD models.^25^ Our findings highlight the robust senomorphic activity of NAM, effectively preventing senescence and suppressing SASP production in ACH‐induced senescent liver cells and ALD mice. Compared with ABT263‐mediated SnC clearance, NAM represents a safer and more effective therapeutic strategy for ALD.

## 2 RESULTS

### 2.1 ACH induces senescence of liver cells

**FIGURE 1:**
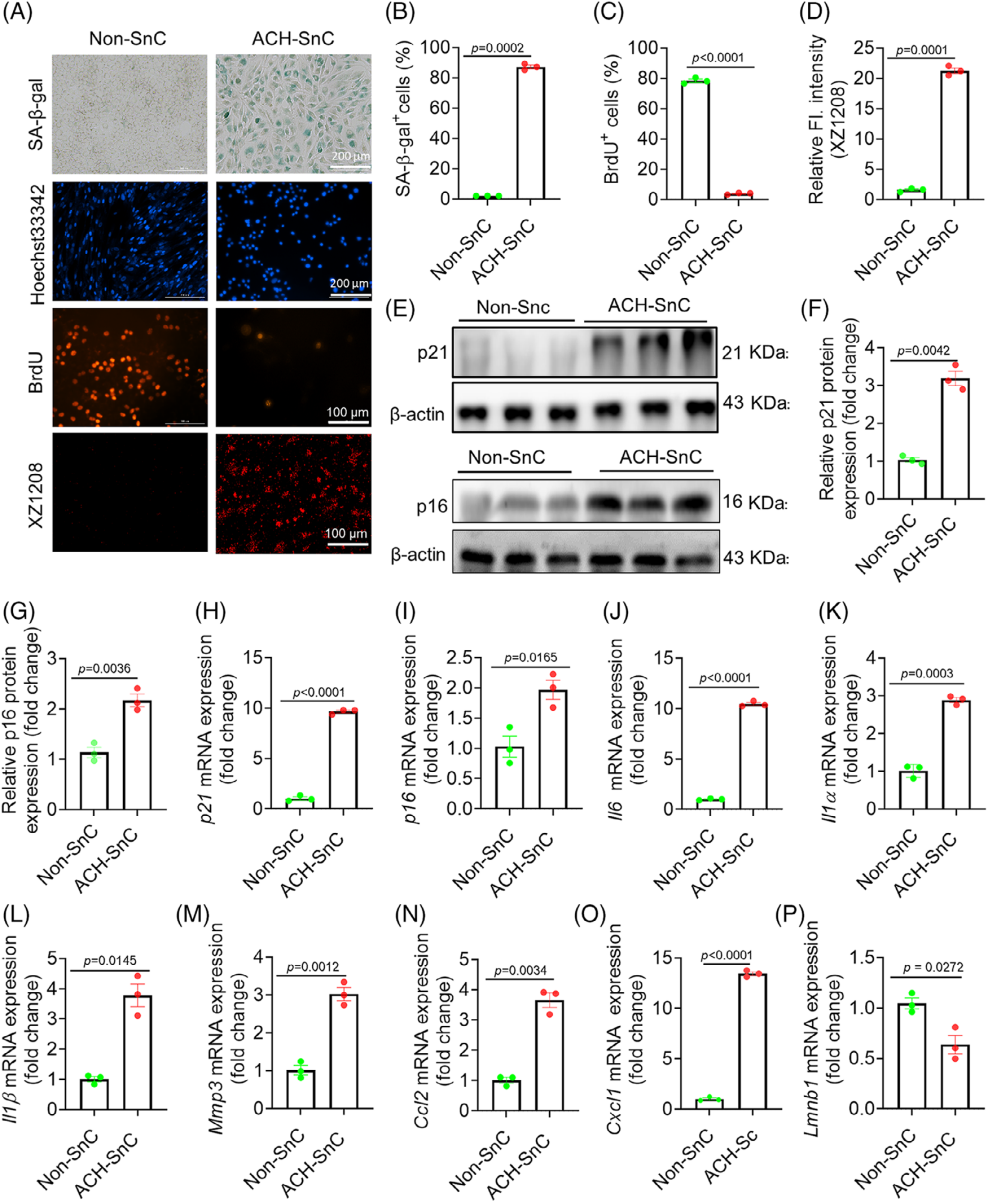
Acetaldehyde (ACH) induces senescence of liver cells. (A) Representative images of SA‐β‐gal (scale bar = 200 µm), Hoechst33342 (scale bar = 200 µm), BrdU (scale bar = 100 µm), and XZ1208 staining (scale bar = 100 µm) of AML12 cells. Cells were treated with ACH for 24 h, then left for 8 d to become senescent before the experiment was performed and the image was taken. (B and C) Quantitative data analysis of SA‐β‐gal staining and BrdU staining in nonsenescent (Non‐SnC) and acetaldehyde‐induced senescent (ACH‐SnC) AML12 cells. (D) Quantification of relative fluorescent intensity (FI) of senescence probe XZ1208 in Non‐SnC and ACH‐SnC AML12 cells. Cells were treated with 10 µM XZ1208 for 24 h (*n* = 3 biologically independent samples). (E) Western blotting assay of p21 and p16 protein levels in Non‐SnC and ACH‐SnC AML12 cells. (F and G) Quantification of p21 and p16 protein levels in Non‐SnC and ACH‐SnC AML12 cells (*n* = 3 biologically independent samples). (H–P) Quantitative analysis of mRNA expressions of *p21* (H), *p16* (I), senescence‐associated secretory phenotype (SASP) factors *Il6* (J), *Il1α* (K), *Il1β* (L), *Mmp3* (M), *Ccl2* (N), and *Cxcl1* (O), and *Lmnb1* (P). Data are presented as mean ± SEM (*n* = 3 biologically independent samples). All statistical analyses presented were done using unpaired t‐tests with Welch's correction.

ACH is the major metabolite of alcohol that causes toxicity to liver cells. To evaluate the therapeutic potential of targeting liver senescence for treating ALD, we established senescent liver cell models using ACH. As hepatocytes and stellates are two major cell types that undergo senescence in liver diseases,^26^ we selected the representative hepatocyte (AML12) and human hepatic stellate (LX2) for senescence model construction. The half‐maximal inhibitory concentration (IC_50_) value of ACH was 3.85 and 2.79 mM for AML12 and LX2 cells, respectively (Figure S1A,B). Sublethal doses of 3.0 and 2.5 mM ACH were used to induce senescence for these two cell lines, respectively. ACH treatment significantly increased the activity of senescence‐associated beta‐galactosidase (SA‐β‐gal) (Figures 1A,B and S1C,D), one of the most widely accepted markers for SnC detection,^27^ compared with the non‐SnCs (Non‐SnCs). Meanwhile, it substantially reduced DNA synthesis indicated by BrdU staining (Figures 1A,C and S1C,E). Using our previously developed senescence‐detecting probe XZ1208,^28^ we found that only ACH‐treated AML12 and LX2 cells can be labeled, showing a bright fluorescent signal (Figures 1A,D and S1C,F), indicating that ACH was able to induce liver cell senescence (named ACH‐SnC).

The mRNA and/or protein expression levels of p16 and p21, two major cell cycle regulators and senescence markers, were significantly elevated in the ACH‐SnCs than the Non‐SnCs (Figures 1E–I and S1G–J). Consistently, ACH‐SnCs expressed higher mRNA levels of SASP markers (*Il6*, *Il1α*, *Il1β*, *Mmp3*, *Ccl2*, and *Cxcl1*) and a lower level of *Lmnb1* (Figure 1J–P). To confirm the senescence phenotypes induced by ACH, we used X‐ray irradiation (IR) to induce the senescence of LX2 cells as positive controls. In comparison with Non‐SnCs, we found upregulated mRNA levels of p21, p16, and SASP factors in IR‐induced SnCs (IR‐SnCs) (Figure S1H–O), paralleling the findings in ACH‐SnCs. Taken together, the increased SA‐β‐gal activity, halted cell proliferation, and increased expression levels of p16, p21, and numerous SASP factors suggest that sublethal exposure to ACH was sufficient to induce senescence in the liver cells. This finding establishes ACH‐SnCs as a reliable senescence model for further study.

### 2.2 ABT263 can selectively kill senescent liver cells induced by ACH

**FIGURE 2:**
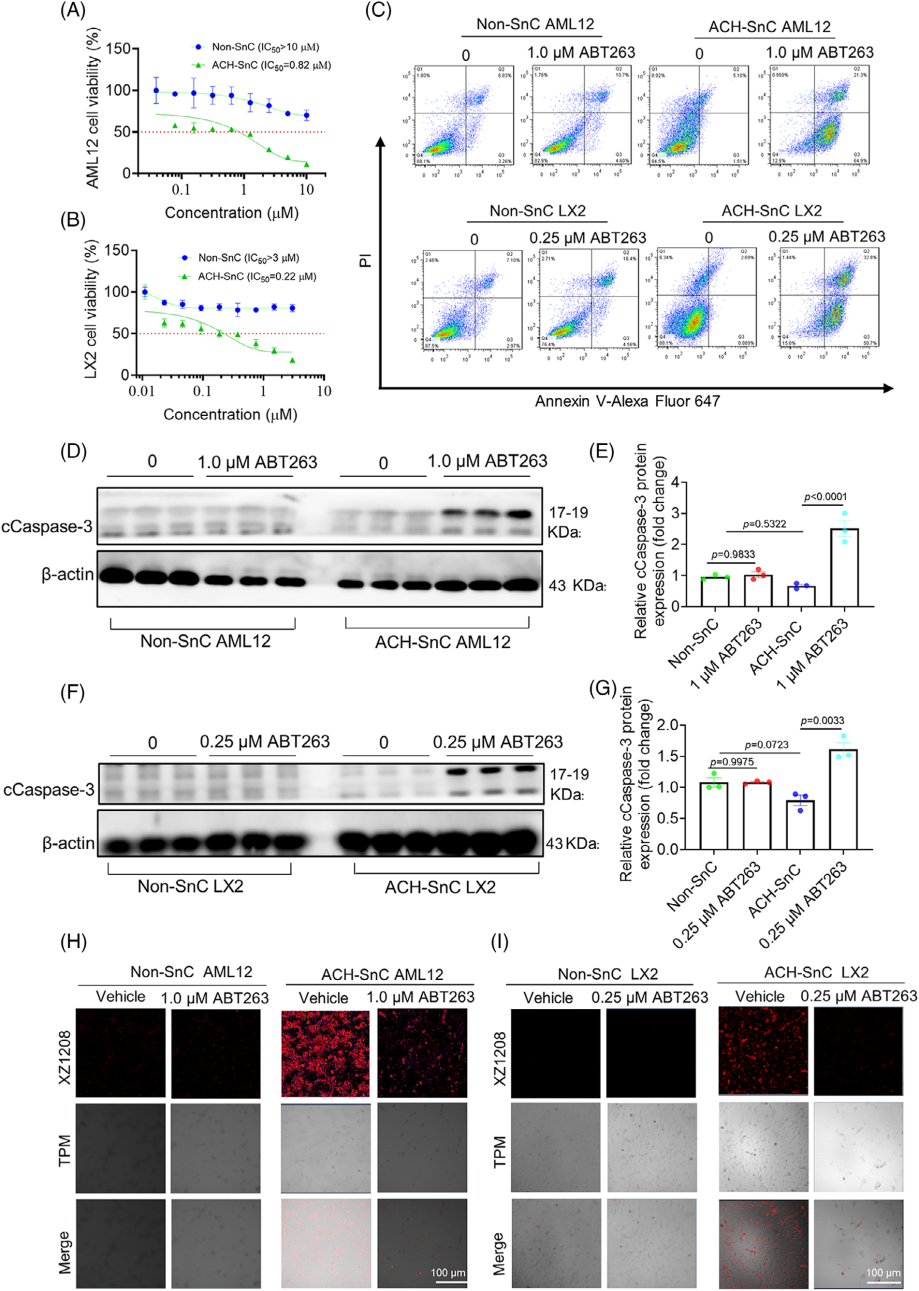
ABT263 selectively kills senescent liver cells. (A and B) MTS assay showing the IC_50_ values in Non‐SnC and ACH‐SnC AML12 and LX2 cells treated with ABT263 for 24 h. (C) Flow cytometry analysis of apoptosis induction in Non‐SnC and ACH‐SnC AML12 and LX2 cells by Annexin‐V and PI staining after treatment with indicated concentrations of ABT263 for 24 h. (D) Western blotting assay of cleaved Caspase‐3 (cCaspase‐3) in Non‐SnC and ACH‐SnC AML12 cells treated with ABT263 for 24 h. (E) Quantitative data analysis of cCaspase‐3 expression in AML12 cells (*n* = 3 biologically independent samples). (F) Western blotting assay of cCaspase‐3 in Non‐SnC and ACH‐SnC LX2 cells treated with ABT263 for 24 h. (G) Quantitative data analysis of cCaspase‐3 expression in LX2 cells (*n* = 3 biologically independent samples). (H and I) Confocal microscopy images of XZ1208 labeled Non‐SnC and ACH‐SnC AML12 cells (H) and LX2 cells (I) after 24 h treatment with ABT263 (scale bar = 100 µm). Data are presented as mean ± SEM and analyzed using one‐way ANOVA with Tukey's post‐hoc test or Dunnett's multiple comparison test.

We have previously shown that ABT263 (also known as navitoclax) was an effective senolytic compound that can selectively kill SnCs via inhibiting the antiapoptosis protein Bcl‐xL and Bcl‐2.^19^, ^28^ To determine whether ABT263 selectively targets senescent liver cells, we treated ACH‐SnC and Non‐SnC AML12 and LX2 cells with increasing concentrations of ABT263 for 24 h. The IC_50_ values of ABT263 for ACH‐SnC AML12 and LX2 cells were 0.82 and 0.22 µM, respectively, while they were >10 and >3 µM for the Non‐SnC counterparts (Figure 2A,B), indicating the potent senolytic activity of ABT263 against ACH‐SnCs. We next used flow cytometry to examine whether the senolytic effect of ABT263 on liver cells was due to apoptosis. As shown in Figure 2C, ABT263 treatment caused higher early apoptotic (Annexin V^+^, PI^−^) and late apoptotic (Annexin V^+^, PI^+^) cell populations in ACH‐SnC AML12 and LX2 cells, but did not affect the cell populations in the Non‐SnC cells. We then measured the level of cleaved caspase‐3 (cCaspase‐3), a key marker of apoptosis,^29^ in ACH‐SnC AML12 and LX2 cells, and found that cCaspase‐3 levels in ABT263‐treated ACH‐SnC AML12 and LX2 cells were significantly higher compared with untreated cells (Figure 2D–G). To further confirm the sole effect of ABT263 on caspase‐3 activation in cell apoptosis, we performed flow cytometry and Western blotting analysis using both Non‐SnC and ACH‐SnC cells. We found that apoptosis and caspase‐3 activation in ACH‐SnC cells could be inhibited by QVD, a pan‐caspase inhibitor (Figure S2A–D), indicating that apoptosis was caspase‐3 dependent. Furthermore, we measured the senolytic effect of ABT263 using the senescence probe XZ1208.^28^ In the Non‐SnC group, no detectable fluorescent signal was observed before and after treatment with ABT263 due to the absence of SnCs (Figure 2H,I). In contrast, a strong fluorescent signal was detected in the ACH‐SnC groups (Figure 2H,I). Following ABT263 treatment, the fluorescent signal was reduced in ACH‐SnC liver cells, indicating the high efficacy of ABT263 in clearing ACH‐SnCs. Herein, we demonstrate that ABT263 has a strong senolytic activity against ACH‐SnC liver cells via inducing caspase3‐dependent apoptosis.

### 2.3 ABT263 effectively clears SnCs in the liver but increases alanine aminotransferase levels in ALD mice

**FIGURE 3:**
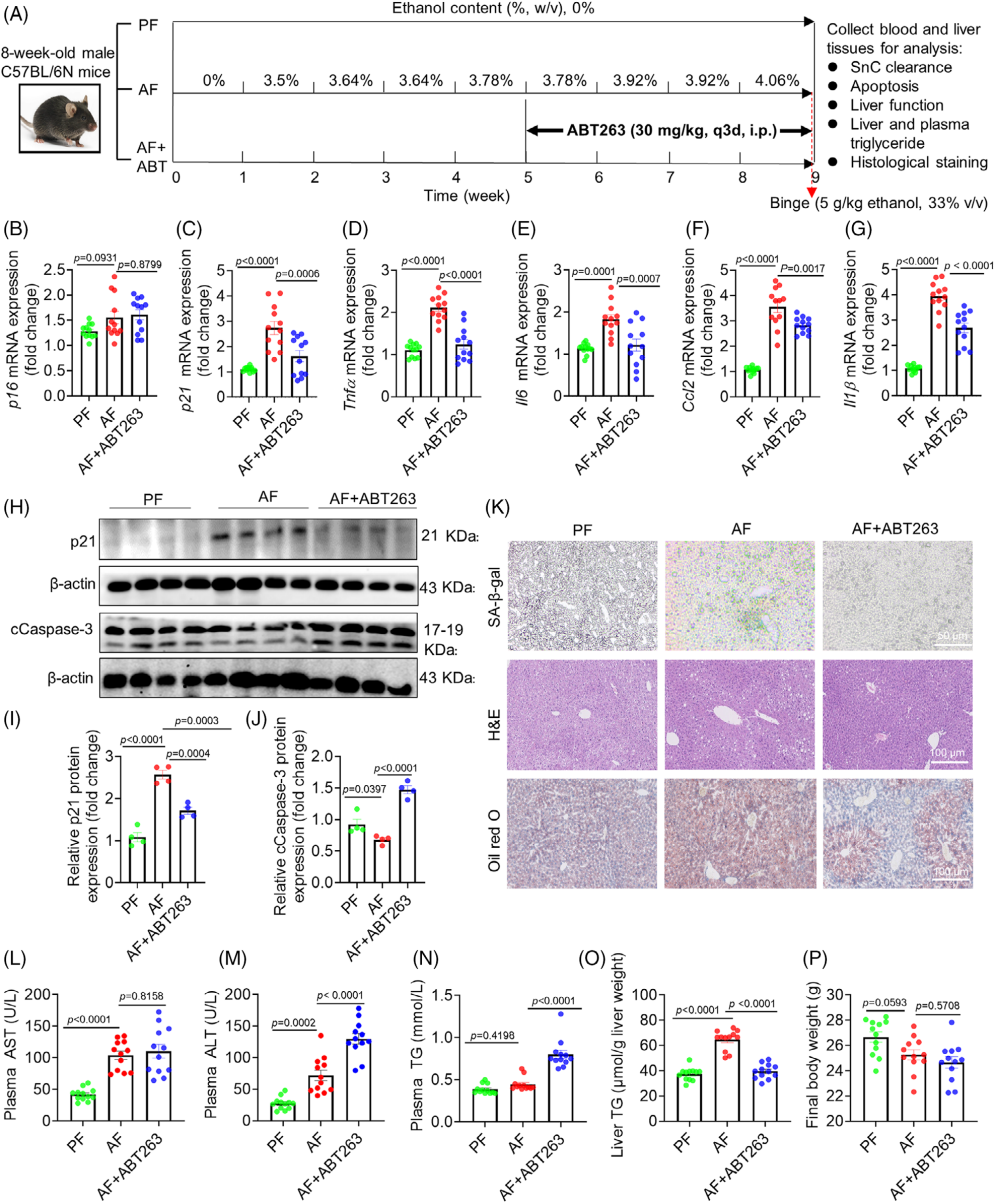
ABT263 effectively clears senescent cells in the liver. (A) Experimental design: 8‐week‐old male mice were fed with the Lieber‐DeCarli diet and divided into pair‐fed (PF), alcohol‐fed (AF), and ABT263‐treated groups (n = 12 mice/group). Mice were fed with ethanol specified in (A) and treated with ABT263 (30 mg/kg, i.p., q3d) from week 5 to 9, totaling 10 injections. After the last treatment, mice received a binge (red arrow) and were euthanized, and tissues were harvested for analysis. (B‐G) mRNA expressions of *p16* (B), *p21* (C), *Tnfα* (D), *Il6* (E), *Ccl2* (F), and *Il1β* (G) were analyzed. (H) Western blotting assay of p21 and cCaspase‐3 in the liver of mice. (I and J) Quantification of p21 and cCaspase3 in the liver of mice. Data are presented as mean ± SEM (*n* = 4 mice/group). (K) Images of SA‐β‐gal staining, H&E staining, and Oil red O staining of liver sections (scale bars = 50 µm for SA‐β‐gal, 100 µm for H&E and Oil red O). (L–N) Analysis of plasma AST (L), ALT (M), and TG (N) in mice. (O and P) Levels of liver (TG) and body weight of mice. Data are presented as mean ± SEM and analyzed using one‐way ANOVA with Tukey's post‐hoc test or Dunnett's multiple comparison test.

We proceeded to examine the effect of clearance of liver SnCs on liver function in an in vivo model of ALD mice. First, we evaluated the effect of ABT263 on liver function and blood parameters in a preliminary study (Figure S3A). At a dose of 30 mg/kg, ABT263 did not cause significant changes in body weight (BW), alanine aminotransferase (ALT), aspartate transaminase (AST), lipids, blood cell counts, major organ weights, and cCaspase‐3 expression (Figure S3B–S), indicating that ABT263 alone did not cause liver injury or significant toxicity in normal mice. In the ALD model, C57BL/6N wild‐type (WT) mice were divided into three groups subjected to the Lieber‐DeCarli diet for 8 weeks: an isoenergetic pair‐fed (PF) group serving as the control, an alcohol‐fed (AF) group, and the alcohol‐fed ABT263 treatment group (AF+ABT263) (Figure 3A) according to our previous protocol.^30^ We measured and compared the mRNA levels of senescence markers *p16*, *p21*, and major SASP markers (*Tnfα*, *Il6*, *Ccl2*, and *Il1β*) in mice livers of all groups. We did not observe any changes in *p16* mRNA or protein levels (Figures 3B and S4A), but found significantly upregulated mRNA levels of *p21* and SASP factors (Figure 3C–G). Consistently, the protein level of p21 was also increased by administration of alcohol (Figure 3H,I). These results suggest that alcohol intake induced liver senescence in a p21‐ rather than p16‐dependent manner. Further, ABT263 treatment significantly decreased SA‐β‐gal activity, likely due to the clearance of liver SnCs via triggering caspase 3‐dependent apoptosis (Figures 3H–K and S4B). These results indicate that ABT263 robustly reduced the ACH‐SnCs by inducing apoptosis in the liver of ALD mice.

We next assessed the effect of ABT263 on the liver function of ALD mice. While AST levels showed no change, plasma ALT and triglyceride (TG) levels were elevated in the ABT263‐treated group compared with the AF group (Figure 3L–N). Interestingly, we observed a significant reduction in liver weight (Figure S4C), which may be associated with reduced TG content in the liver, as demonstrated by Oil red O staining and TG quantification (Figure 3K,O). Whole‐body and most organ weights remained unchanged across groups (Figures 3P and S4D–G) except for a slight reduction in kidney weight observed in the AF group, which was restored following ABT263 treatment (Figure S4H). These results demonstrate that ABT263 effectively removed SnCs and reduced lipid accumulation in the liver of ALD mice, but increased liver injury indicator levels.

### 2.4 NAM suppresses senescence and SASP in liver cells

Given that ABT263 successfully cleared senescent liver cells, but unexpectedly increased ALT levels, we hypothesized whether suppression of senescence or SASP (senomorphic effect) without killing SnCs can protect the liver from alcohol‐induced injury. NAM, which is involved in the biosynthesis of NAM adenine dinucleotide (NAD), an essential coenzyme critical for maintaining redox balance and cellular metabolism, was used to treat liver cells and mice. As NAM has been shown to alleviate SnC accumulation in mesenchymal stem cells,^31^ we investigated whether NAM could suppress senescence and SASP in the liver of mice fed with alcohol.

**FIGURE 4:**
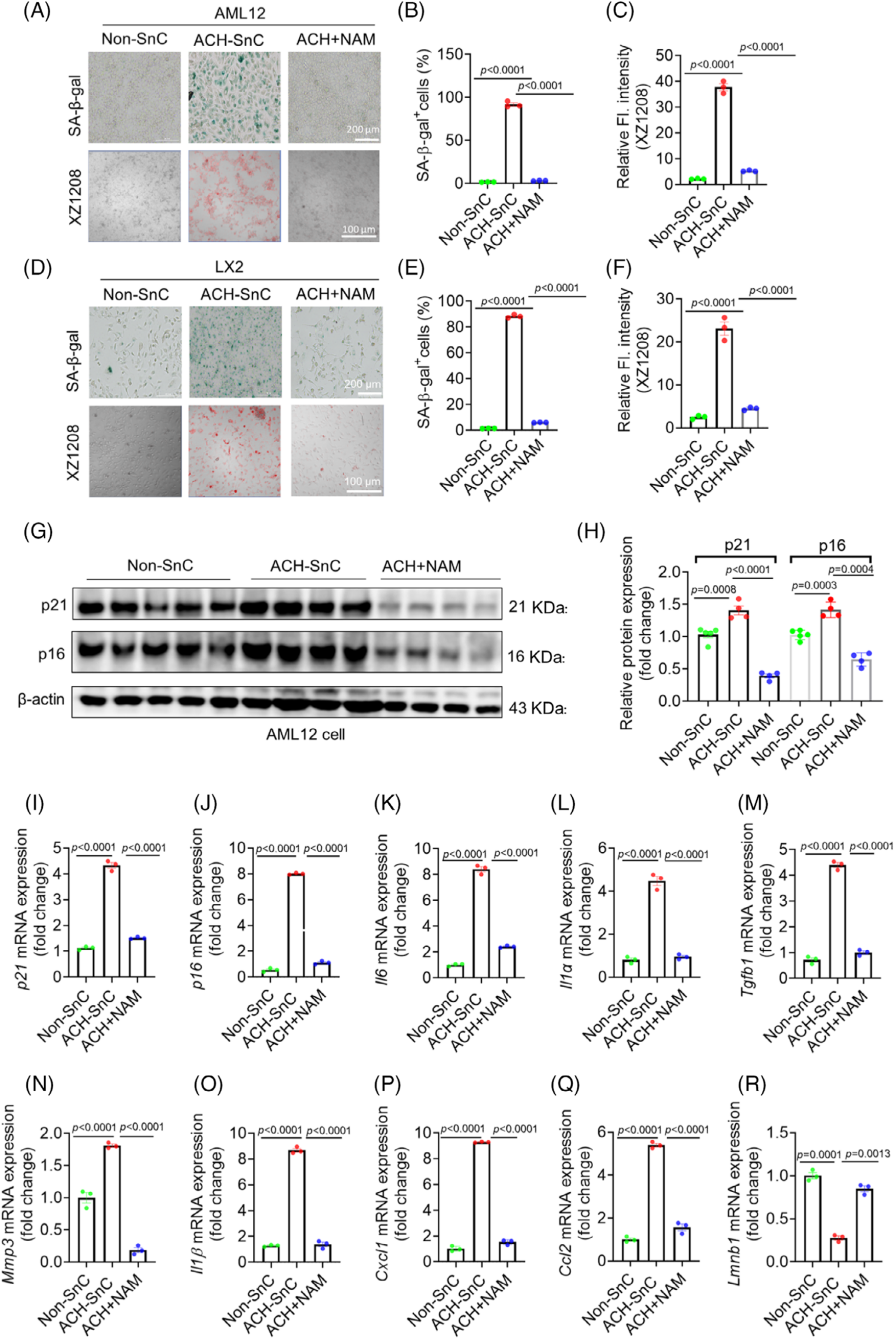
NAM suppresses senescence and senescence‐associated secretory phenotype (SASP) in liver cells. (A–C) Images of SA‐β‐gal and fluorescent signal of XZ1208 in non‐senescent (Non‐SnC) or acetaldehyde (ACH) induced senescent cells (ACH‐SnC) (scale bar = 200 µm). AML12 cells were treated with ACH for 24 h, followed by treatment with NAM for 24 h, and then left for 8 d to become senescent. 10 µM XZ1208 was used to stain Non‐SnC and ACH‐SnC AML12 cells (scale bar = 100 µm). (D–F) The same experiments were done in LX2 cells as in AML12 cells. (G‐H) Western blotting analysis of p21 and p16 expression in AML12 cells (*n* = 5, 4, and 4 independent samples, respectively). (I–R) mRNA expressions of *p21* (I), *p16* (J), and the SASP factors *Il6* (K), *Il1α* (L), *Tgfb1* (M) *Mmp3* (N), *Il1β* (O), *Cxcl1* (P), *Ccl2* (Q), and *Lmnb1* (R). Data are presented as mean ± SEM (n = 3 biologically independent samples), and analyzed using one‐way ANOVA with Dunnett's multiple comparison test.

We first tested the effect of NAM on cell viability of liver cells, and their IC_50_ values were at millimolar levels in both AML12 and LX2 cells (Figure S5A,B), suggesting that they are relatively less toxic to liver cells. Interestingly, ACH‐SnC liver cells were more resistant to NAM compared with Non‐SnC liver cells (Figure S5A,B). We next used ACH‐SnC models to initially evaluate the role of NAM in senescence and SASP. We observed more SA‐β‐gal staining positive cells with drastically higher mRNA and protein levels of senescence markers p16 and p21, and higher mRNA levels of SASP in ACH‐SnC AML12 and LX2 cells (Figures 4A–Q and S5C–N). Immediate incubation of NAM for 24h after 24 h treatment of ACH can block liver cell senescence, as indicated by reduced SA‐β‐gal activity, fluorescent senescence signal, and mRNA/protein of p16, p21, and SASP factors in the NAM‐treated group compared with the ACH‐SnC group (Figures 4A–Q and S5C–N). Loss of lamin B1 was identified as another major senescence biomarker that is often reduced in SnCs, notably at the mRNA level through decreased mRNA stability.^32^ We found a significant reduction of *Lmnb1* in the ACH‐SnCs compared with the Non‐SnC control, which can be restored by treatment of NAM (Figure 4R). These results collectively suggest that NAM is highly effective at suppressing ACH exposure‐induced senescence and SASP in liver cells.

### 2.5 NAM prevents liver injury induced by alcohol consumption in ALD mice

**FIGURE 5:**
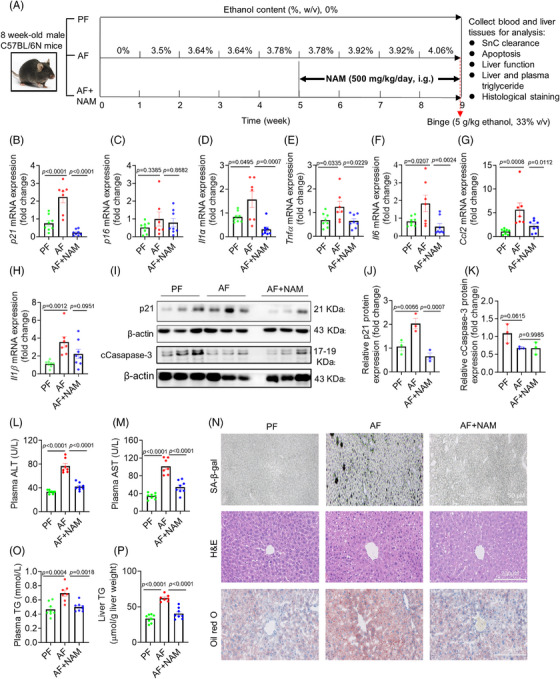
NAM mitigates liver injury induced by alcohol consumption in mice fed with alcohol. (A) Experimental design: 8‐week‐old mice were fed with Lieber‐DeCarli diet and divided into pair‐fed (PF), alcohol‐fed (AF), and NAM‐treated groups (*n* = 8, 7, 8 mice/group, respectively). Mice were treated with NAM (500 mg/kg every day, i.g.) from week 5 to 9, received a binge (red arrow), and were euthanized after the last treatment to harvest various tissues for analyses. (B–H) Quantitative analysis of mRNA expressions of *p21* (B), *p16* (C), *Il1α* (D), *Tnfα* (E), *Il6* (F), *Ccl2* (G), and *Il1β* (H) in the liver. (I) Western blotting analysis of p21 and cCaspase‐3. (J and K) Quantification of the expression of p21 and cCaspase‐3 in the liver of PF, AF, and NAM‐treated mice (*n* = 3 mice/group. (L) Analysis of blood biochemical indicators of plasma ALT (L) and AST (M). (N) Representative images of SA‐β‐gal (scale bar = 50 µm), H&E (scale bar = 200 µm), and Oil red O staining (scale bar = 200 µm) of liver sections of mice. (O and P) Plasma and liver triglyceride (TG) in PF, AF, and NAM‐treated AF mice. Data are presented as mean ± SEM and were analyzed using one‐way ANOVA with Dunnett's multiple comparison test.

The promising in vitro findings suggest that NAM may be a superior option for suppressing senescence phenotype with a low risk of aggravating liver injuries in ALD mice. Our preliminary study showed that NAM alone did not cause toxicities in normal mice (Figure S3A–Q). We conducted an ALD mouse experiment as illustrated in Figure 5A, and found that the mRNA levels of *p21* were markedly increased in the AF mice but reduced in the NAM group (Figure 5B). Again, there were no significant changes in p16 across all groups (Figure 5C), suggesting that alcohol‐induced liver senescence was dependent on p21 instead of p16. Consistently, the mRNA levels of *p21* and main SASP markers were significantly reduced by the treatment of NAM compared with the AF group (Figure 5B, D–H). Western blotting results further confirmed the suppressive role of NAM on senescence marker p21 (Figure 5I–J). In addition, NAM supplementation did not induce significant changes in the apoptosis marker compared with that in AF group (Figure 5I,K). These findings suggest excellent suppression of senescence and SASP by NAM treatment. Of note is that NAM treatment cannot reverse senescence or inhibit SASP if it was administrated after cells become senescent completely (Figure S6A–I).

We then evaluated the liver function across all mice groups. Plasma ALT and AST levels were significantly higher in the AF group compared with the PF group, which can be completely suppressed by NAM treatment (Figure 5L,M), in line with the changes in SA‐β‐gal activity (Figure 5N) and other senescence marker levels (Figure 5B, D–J). Consistently, NAM can effectively reduce plasma and liver TG content compared with the AF group (Figure 5O–P), without affecting liver tissue architecture as indicated by hematoxylin and eosin (H&E) staining (Figure 5N). To summarize, these results demonstrate that NAM robustly protected mice from alcohol‐induced liver injury. The protective effect was possibly mediated by suppression of senescence and SASP in the liver.

### 2.6 ABT263 and NAM play different roles in regulating gene expressions in the liver of ALD mice

**FIGURE 6:**
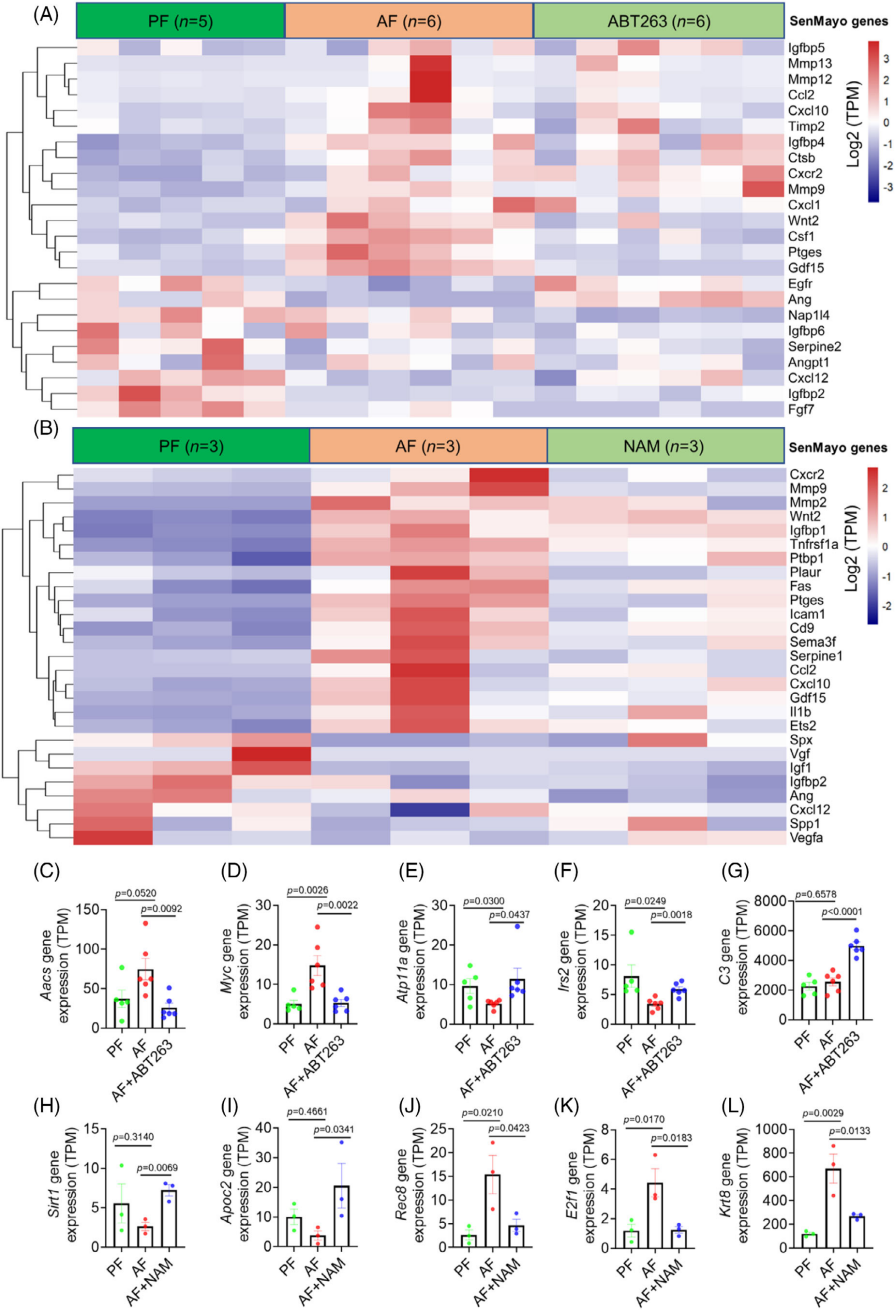
RNA‐seq analysis on the liver of alcoholic disease mice treated with ABT263 or nicotinamide (NAM). (A and B) Heatmap of the senescence‐associated gene (SenoMayo genes) expressions in the liver of mice fed with isogenic diet (PF), alcohol‐fed (AF) in ABT263 (A) or NAM (B) treated mice. (C–G) Representative differentially expressed genes (DEGs) in the liver of mice treated with ABT263 (*n* = 5, 6, 6 mice/group, respectively). (H–L) Representative DEGs in the liver of mice treated with NAM (*n* = 3 mice/group). Gene expression levels were indicated by transcripts per million (TPM). Data are presented as mean ± SEM and analyzed using one‐way ANOVA with Tukey's post‐hoc tests or Dunnet's post‐hoc test.

To explore the clues for why ABT263 and NAM differentially regulated liver function in the ALD mice, we performed RNA‐sequencing (RNA‐seq) analysis to identify differentially expressed genes (DEGs) in the liver of ALD mice treated with ABT263 and NAM, respectively (Figure S7A). Compared with the PF group, alcohol intake significantly upregulated 1048 genes, which were enriched in the cellular response to xenobiotic stimulus, xenobiotic catabolic process, chemical carcinogenesis, and so on (Figure S7B). The 1293 downregulated genes were enriched in alcohol and mRNA metabolic processes and PPAR signaling pathways (Figure S7C). To explore whether the DEGs are associated with senescence, we retrieved a panel of 119 senescence‐associated genes termed SenoMayo.^33^ Among them, 43 DEGs are in the list of SenoMayo genes (Figure 6A,B and Table S1), and some have been validated by quantitative polymerase chain reaction (qPCR) in cells or liver tissues, such as *Cxcl1*, *Cxcl12*, and *Ccl2*. These data suggest that ethanol intake induces senescence in mice liver. Interestingly, some SenoMayo genes were significantly downregulated by ABT263 treatment (Figure 6A), and the other downregulated genes were enriched in xenobiotic catabolic process, cellular response to xenobiotic stimulus, and biological oxidations (Figures 6A and S8A,B). Furthermore, ABT263 treatment upregulated 260 genes related to the alcohol metabolic process, and binding/uptake of ligands by scavenger receptors (Figure S8A,B). Exploring the DEGs, we found that ABT263 downregulated lipogenesis‐related genes (e.g., *Aacs* ^34^ and *Myc* ^35^), but upregulated some genes for energy metabolism (e.g., *Atp11a* and *Irs2* ^36^) (Figure 6C–F). However, ABT263 activated complement cascade via upregulating complement component genes, such as *C3* (Figures 6G and S8E), which may aggravate liver injury induced by alcohol.^37^

In contrast, NAM treatment significantly upregulated 274 genes enriched in damage repair (UV‐damage excision repair and double‐strand break repair), mRNA metabolism, RNA splicing, and so on, and downregulated 143 genes enriched in type 1 interferon (IFN)‐mediated signaling pathway, antiviral mechanism by IFN, and apoptotic signaling, and so on (Figure S8C,D). Among the DEGs, we observed that NAM upregulated some key genes in metabolism, for example, *Sirt1* and *Apoc2* (Figures 6H,I, and S8F), and downregulated 27 senescence‐associated genes (Figure 6B) and some genes acting as DNA damage markers, for example, *Rec8*, *E2f1*, and *Krt8* (Figure 6J–L).

Alcohol‐induced liver injury is modulated by NOD‐like receptor inflammasomes.^38^ As ABT263 increased the ALT levels in the ALD model, we tested IL1β, the downstream effective protein of inflammasomes, to see whether the increased ALT was associated with inflammasome activation. Results show that ethanol intake did not change the pro‐IL1β or IL1β levels in the liver, while ABT263 reduced the protein levels of pro‐IL1β (Figure S9A). Due to the proinflammatory necrosis in the liver injury,^39^ we further tested the key effector protein responsible for necrosis. Consistently, ABT263 reduced the marker of liver necrosis as indicated by reduced phosphorylation of MLKL (Figure S9B). These data suggest the dual roles of ABT263 in reducing liver TG content but increasing ALT and plasma TG levels, which may not be associated with inflammasome formation or liver necrosis. In summary, the above data suggest that targeting liver senescence can be a new approach to treat ALD. Compared with clearance of senescent liver cells, suppression of their senescence could be a better way for ALD treatment.

## 3 DISCUSSION

This study demonstrates that targeting cellular senescence offers a promising therapeutic approach for ALD. We explored both senolytic and senomorphic strategies by utilizing ABT263, a potent senolytic agent, and NAM, a senomorphic NAD^+^ precursor, to address the senescence burden in ALD. While ABT263 effectively cleared ACH‐induced SnCs in vitro and in vivo, it was associated with elevated liver injury markers, suggesting a potential trade‐off between SnC clearance and liver function preservation. Conversely, NAM exhibited a robust capacity to suppress senescence and the SASP in liver cells, reducing liver injury markers and improving metabolic function in ALD mice. These findings highlight distinct mechanisms of action between senolytics and senomorphics, with NAM showing potential as a safer and more effective therapeutic option for managing ALD.

Cellular senescence has been implicated in a wide range of age‐related diseases, including chronic liver diseases such as NAFLD and ALD.^13^, ^16^, ^40^, ^41^ Senescence‐related inflammatory pathways contribute to liver dysfunction and progression of ALD, exacerbated by alcohol metabolism. Previously, alcohol‐induced liver injury was attributed primarily to oxidative stress caused by alcohol metabolism in the liver, where alcohol dehydrogenase and ACH dehydrogenase produce ACH as a toxic byproduct.^8^, ^42^ This process disrupts lipid metabolism, leading to hepatic steatosis and cholesterol accumulation, which can accelerate ALD progression.^43^ Recent evidence, however, links alcohol‐induced senescence in hepatocytes to liver dysfunction and ALD progression, showing that alcohol intake downregulates protective genes such as Sirt1, while activating pro‐senescent pathways like NFATc4 and repressing antisenescent pathways like PPARγ.^44^, ^45^ Senescent liver cells, characterized by mitochondrial dysfunction, inefficiently metabolize fatty acids, further contributing to steatosis.^15^ Our findings confirm that ACH induces liver cell senescence, marked by increased expression of senescence markers p16 and p21 and SASP factors such as Il6 and Mmp3, thereby establishing ACH‐SnCs as a valid model of ALD. Targeting these SnCs with either senolytic or senomorphic agents offers therapeutic potential for ALD. Senolytics such as ABT263 aim to clear SnCs, while senomorphics like NAM suppress the senescence phenotype without inducing apoptosis, addressing the inflammation and cellular dysfunction without risking the release of injury markers associated with SnC clearance.

ABT263 demonstrated potent senolytic activity by selectively inducing apoptosis in ACH‐SnCs, reducing SA‐β‐gal activity, and lowering TG content in the liver. These effects align with previous studies showing that clearance of SnCs can mitigate hepatic steatosis and inflammation, as seen in NAFLD models where senolytic therapy reduced liver fat accumulation.^15^ The effect of ABT263 on adipogenesis‐related genes such as Aacs and Myc, along with its upregulation of energy metabolism genes like Atp11a and Irs2, likely contributed to the observed reduction in hepatic TG content. However, senescent hepatic stellate cells (HSCs) can promote liver regeneration via secretion of IL‐6 and CXCR2 ligands.^46^ Clearance of senescent HSCs might inadvertently impair the liver's ability to renew and repair, potentially explaining the rise in liver injury markers. Additionally, ABT263's activation of the complement cascade in the liver, as indicated by the upregulation of genes (such as C3), may have further contributed to inflammation and liver damage in ALD.^37^ One consideration in using ABT263 is its mechanism of action, which involves the inhibition of antiapoptotic proteins like Bcl‐xL and Bcl‐2, known to induce platelet apoptosis and lead to thrombocytopenia.^47^ Although our study used a low dose of ABT263 that did not affect platelet count, further investigation is required to determine if the observed hepatotoxicity, including elevated ALT levels, is related to ABT263's on‐target effects in ALD.

The efficacy of NAM as a senomorphic agent offers a safer alternative to senolytic therapy in ALD. Unlike ABT263, NAM did not induce apoptosis but instead suppressed senescence and SASP in both in vitro and in vivo models, demonstrating a strong protective effect against alcohol‐induced liver injury. The mechanism of NAM involves restoration of NAD^+^ levels, which supports mitochondrial function and SIRT1 activity, crucial for redox balance, lipid metabolism, and reducing oxidative stress.^48^, ^49^, ^50^ The upregulation of Sirt1 by NAM aligns with improved liver function and decreased markers of liver injury, suggesting that NAM mitigates mitochondrial dysfunction and inflammation without the adverse effects associated with SnC clearance. While the therapeutic benefits of NAM are promising, its potential side effects, particularly at high doses, warrant careful consideration. For instance, NAM has been reported to decrease insulin sensitivity and inhibit poly(ADP‐ribose) polymerases, which may affect genome stability and cancer risk.^51^, ^52^, ^53^, ^54^ Therefore, while NAM shows potential as a treatment for ALD, further studies are needed to determine safe dosing strategies and to evaluate long‐term effects in patients.

In our study, ACH‐SnC hepatocytes and HSCs exhibited elevated levels of p16 and p21, markers of senescence, while in the ALD mouse model, only p21 and SASP factors were upregulated, with no significant changes observed in p16 expression. This finding is consistent with previous research in liver injury models, such as fumarylacetoacetate hydrolase (Fah) knockout mice and partial hepatectomy models, where p21 is a more prominent marker of liver senescence than p16.^55^, ^56^ These observations suggest that p21 may serve as a more reliable indicator of senescence in mouse models of liver injury, providing insights for future studies on senescence‐targeted therapies in ALD. Our RNA‐seq analysis provided valuable insights into the distinct molecular mechanisms by which ABT263 and NAM exert their effects on the liver in ALD. ABT263 treatment downregulated genes involved in lipogenesis and upregulated genes associated with complement activation, supporting its dual effects on reducing liver TG while increasing ALT level. On the other hand, NAM upregulated genes related to DNA repair and downregulated IFN‐mediated signaling pathways, suggesting a reparative role in ALD. Additionally, NAM's upregulation of *Sirt1*, a key regulator of metabolism and stress resistance, supports its role in modulating hepatic responses to alcohol‐induced injury. The differential gene expression profiles induced by ABT263 and NAM underscore the complexity of senotherapeutic interventions in ALD. While ABT263 may have a role in reducing lipid accumulation, its activation of complement pathways could limit its utility in chronic ALD management. NAM, by contrast, appears to engage protective metabolic and reparative pathways that reduce senescence and SASP without inducing apoptosis or inflammation, offering a more balanced approach to treating ALD. The limitations observed with ABT263 treatment underscore the challenges of senolytic therapy in ALD. Although ABT263 has shown promise in clearing SnCs, the associated side effects suggest that it may not be ideal as monotherapy for ALD. Additional studies are warranted to further elucidate the balance between the beneficial effects of SnC clearance and the potential for off‐target impacts on liver function in the context of chronic alcohol exposure.

Our results suggest that NAM, with its ability to suppress senescence and SASP without triggering apoptosis, represents a safer therapeutic strategy for managing ALD than senolytic agents like ABT263. While ABT263 may offer benefits by reducing SnC burden and improving lipid metabolism, their potential to elevate liver injury markers poses a risk for chronic ALD management. Further investigation into the long‐term efficacy and safety of NAM, as well as combination therapies that might leverage the benefits of both senomorphic and senolytic approaches, could advance ALD treatment.

In summary, our study provides compelling evidence for two therapeutic strategies targeting liver senescence in ALD. The senomorphic effect of NAM is promising, suggesting that senescence suppression may offer a safer and more effective approach to mitigate ALD progression compared with the selective clearance of senescent liver cells by ABT263. Continued research into senotherapeutics will be essential for developing optimized treatments for ALD and related liver diseases.

## 4 MATERIALS AND METHODS

### 4.1 Cell culture

The hepatic cell lines AML12 and LX2 were used in this study due to their significant roles in liver function and disease, notably in the context of alcohol‐induced liver injury.^57^, ^58^ AML12 cells are mouse hepatocytes purchased from the Kunming Cell Bank of Type Culture Collection (Kunming, China). LX2 cells are human HSCs gifted by Professor Bin Liang from Yunnan University. AML12 and LX2 cells were authenticated by short tandem repeat (STR) sequencing performed by Tsingke Biotechnology (Beijing, China). All cells were cultured in complete Dulbecco's modified eagle medium (C11995500BT; Gibco, MD, USA) supplemented with 10% fetal bovine serum (RY‐F22; Royacel Biotechnology Co., Ltd., Lanzhou, China) and 1% penicillin/streptomycin (15140‐122; Gibco) in a humidified incubator at 37°C and 5% CO_2_.

### 4.2 Senescence induction

Senescence was induced by ACH exposure or IR. ACH (75‐07‐0; Sigma–Aldrich, St. Louis, MO, USA) was administered at pre‐determined concentrations (3.0 mM for AML12 cells and 2.5 mM for LX2 cells) for 24 h. Following ACH exposure, cells were allowed to recover for 3 d before undergoing a single passage at a ratio of 1:3. Notably, both AML12 and LX2 cell lines exhibited complete senescence within 10 d postinduction, as indicated by permanent growth arrest and a series of senescence markers. For IR‐induced senescence, cells were cultured in a 100 mm cell plate and irradiated at 300 kV, 10 mA for 8 min and incubated for 24 h at 37°C. After 10 d, cells become senescent, which were confirmed by observing morphological changes and determining senescence markers, such as DNA synthesis, mRNA levels of *p16*, *p21*, *Lamnb1*, and SASP factors.

### 4.3 Cell treatment with NAM or ABT263

AML12 and LX2 cells were cultured in a 100 mm cell culture plate. This ensured optimal cell density for subsequent experiments. To induce senescence, we exposed the AML12 cells to 3.0 and 2.5 mM ACH respectively for 24 h. After ACH exposure, the medium containing ACH was carefully removed. Fresh medium supplemented with 0.5 mM NAM was added to the cells. Cells were then incubated for an additional 24 h to allow for the uptake and utilization of these compounds. Following the NAM treatment, the medium containing these compounds was replaced with fresh culture medium. After a total duration of 12 d (including the induction phase and the NAM treatment days), the cells were harvested for further experiments.

Before ABT263 treatment, cells were induced senescent as described above. Simply, cells were treated with ACH for 24 h, then the medium containing ACH was removed and fresh new medium was added and incubated for 8–10 d to become senescent. 1 and 0.25 µM of ABT263 (923564‐51‐6; GlpBio Technology, CA, USA) were then used to treat ACH‐SnC AML12 and LX2, respectively for 24 h, after which the cells were collected for further analysis.

### 4.4 SA‐β‐gal staining

SA‐β‐galactosidase staining was employed to identify SnCs following ACH treatment. This assay leverages the detection of SA‐β‐gal activity at a suboptimal pH of 6.0, a characteristic feature of SnCs.

#### 4.4.1 Cellular SA‐β‐gal staining

Both AML12 and LX2 cells treated with ACH were subjected to the SA‐β‐gal staining protocol (C0602; Beyotime Biotech, Shanghai, China) according to the manufacturer's instructions. Briefly, ACH‐SnCs and Non‐SnCs were cultured in 6‐well plates for 24 h. Cells were then washed with phosphate‐buffered saline (PBS) for 3 min, followed by fixation with the provided fixative solution for 15 min at room temperature. After fixation, cells were incubated in the staining working solution overnight at 37°C. Stained cells were visualized using the Cytation 5 cell‐imaging multi‐mode plate reader (BioTek, Winooski, VT, USA).

#### 4.4.2 Tissue SA‐β‐gal staining

Tissue SA‐β‐gal staining was performed on liver tissue samples using the Senescence β‐galactosidase Staining Kit per the manufacturer's protocol. Stained tissues were visualized using the Axio Observer3 microscope equipped with the Airyscan platform (Carl Zeiss Meditec AG, Jena, Germany).

### 4.5 Quantitative polymerase chain reaction

Reverse transcription qPCR was performed to determine the mRNA expression levels of *p16*, *p21*, and SASP markers in AML12 and LX2 cells, as well as liver tissues isolated from C57BL/6N mice as described previously.^28^

### 4.6 Western blotting

To assess the protein expression levels of key signaling molecules associated with apoptosis and cellular senescence, Western blotting analysis was performed as described previously.^59^ Primary antibody p16 (ab51243) was purchased from Abcam (Cambridge, UK). Other antibodies p21 (37543s and 2947), cCaspase‐3 (9661s), cleaved‐IL1β (83186), phospho‐MLKL (91689), and MLKL (37705) were from Cell Signaling Technology (Danvers, MA, USA).

### 4.7 Oil Red O staining

Oil Red O staining was employed to evaluate lipid accumulation within liver tissue sections isolated from C57BL/6N mice fed with alcohol.

#### 4.7.1 Tissue preparation

Liver tissue sections (5 mm) were prepared for Oil Red O staining. Frozen sections were first washed twice with PBS to remove residual storage media. Subsequently, the sections underwent incubation in 60% isopropanol for 1 min to permeabilize cell membranes. Following permeabilization, the tissues were meticulously dried in a 37°C incubator for approximately 10 min to optimize Oil Red O staining efficiency.

#### 4.7.2 Oil Red O staining solution

The Oil Red O staining solution was freshly prepared for each experiment. Briefly, 0.35 g of Oil Red O powder (C0157S; Beyotime Biotech) was dissolved in 100 mL of 100% isopropanol. This solution was then diluted 1.7‐fold with deionized water for optimal staining intensity and promptly filtered using a syringe filter to eliminate undissolved particles.

#### 4.7.3 Oil Red O staining procedure

Histological slides were meticulously prepared for Oil Red O staining. The slides were initially incubated with the prepared Oil Red O solution for a precise duration of 15 min to allow for specific lipid droplet staining. The Oil Red O solution was then carefully aspirated from the slides. To remove unbound Oil Red O, the slides were washed with 60% isopropanol for several minutes. Following this step, the slides underwent thorough washing with PBS to eliminate residual isopropanol. Next, the slides were briefly counterstained with hematoxylin for 30 s to visualize nuclei. After hematoxylin staining, additional washes were performed using distilled water to remove excess stain. Finally, the prepared slides were mounted using a specialized mounting medium (glycerol in PBS, ratio 6:1) for optimal tissue preservation and visualization. The mounted slides were then analyzed using microscopy.

### 4.8 Live cell fluorescence imaging and quantification

XZ1208, a near‐infrared fluorescent probe exhibiting high specificity and sensitivity for SnCs, was employed for both in vitro and in vivo labeling as described previously.^28^

### 4.9 Cell viability

The MTS assay was employed to assess cell viability, proliferation, and cytotoxicity of drugs or chemicals in vitro. Briefly, 3,000 cells were counted using an automated cell counter and seeded in 96‐well plates. Subsequently, ACH was added to achieve the highest concentration of 100 µM, and cells were further incubated for 24 h at 37°C. Cell viability was determined using the CellTiter 96^®^ AQueous One Solution Cell Proliferation Assay (G1111; Promega, Madison, WI, USA) according to the manufacturer's instructions. The resulting data were used to generate a graph, and the half‐maximal effective concentration (IC_50_) was calculated using GraphPad Prism software version 8.0 (San Diego, CA, USA). This procedure was repeated to assess the cytotoxicity of ABT263 and NAM on both Non‐SnC and SnC AML12 and LX2 cells. The IC_50_ values were determined using GraphPad Prism software and used throughout the experiment.

### 4.10 Cell apoptosis

Apoptosis was performed as previously described.^28^ Briefly, Non‐SnC and ACH‐SnC AML12 and LX2 cells were treated with designated concentrations of ABT263 for 24 h at 37°C. Following incubation, the cells were harvested using polystyrene round‐bottom tubes. Additionally, ACH‐SnC and Non‐SnCs were pretreated with 10 µM QVD for 30 min, after which they were treated with ABT263 for 24 h. Cells were then stained using the Annexin V‐FITC apoptosis detection kit (C1062S; Beyotime Biotech) according to the manufacturer's instructions. The staining protocol involved incubation in the dark at room temperature (20–25°C) for 10–20 min. Apoptotic cells were subsequently quantified using flow cytometry analysis on an LSR Fortessa flow cytometer (Becton Dickinson, CA, USA).

### 4.11 H&E staining

Histological analysis of liver tissue sections was performed using hematoxylin and eosin (H&E) staining as described previously.^30^

### 4.12 BrdU staining

DNA synthesis was evaluated using 5‐ethynyl‐2′‐deoxyuridine (EdU) labeling. Briefly, AML12 and LX2 cells were incubated with EdU using the Cell‐Light EdU Apollo^®^ 567 In Vitro Kit (C10310‐1; Ribobio, Guangzhou, China) according to the manufacturer's instructions. Cells were imaged with the Cytation 5 cell‐imaging multi‐mode plate reader (BioTek).

### 4.13 Animals and their treatment

WT C57BL/6N mice were kept in ventilated cages and under specific pathogen‐free conditions. The Institutional Animal Care and Use Committee of Zhejiang Chinese Medical University approved the animal experimental protocols (approval number 20220919–27). For the compounds, we administered NAM at a dose of 500 mg/kg BW in mice via oral gavage (i.g.), which led to a linear and rapid increase in NAD^+^ levels in the liver.^60^ In a previous study, we used ABT263 at 40 mg/kg over seven doses to clear SnCs in mice.^19^ For this study, we adjusted the ABT263 dose to 30 mg/kg over 10 doses to reduce its potential toxicity. In the NAM batch of the in vivo model, male WT C57BL/6N mice at 8‐week‐old were divided into three groups (n = 7–8/group) and were subjected to either Lieber‐DeCarli alcohol liquid diet (AF) or isoenergetic pair‐feed liquid diet (PF) for 8 weeks plus 1 binge with or without NAM (500 mg/kg BW/d). In the ABT263 batch, following the preceding protocol, male WT C57BL/6N mice at 8‐week‐old were divided into three groups (*n* = 12/group) and were subjected to either a Lieber‐DeCarli AF or PF for 8 weeks plus 1 binge with or without ABT263 (30 mg/kg/q3d, 10 injections, i.p.). In the preliminary study, 8‐week‐old C57BL/6N mice were divided into VEH, NAM, and ABT263 groups (*n* = 6/group). Mice were fed with a normal diet and were treated with VEH (saline), NAM (500 mg/kg BW, i.p. every day), and ABT263 (30 mg/kg, i.p., q3d) for 1 month. The animal feeding patterns followed those established in a previous study.^30^ Briefly, after 1 week of liquid chow acclimatization in mice, the ethanol concentrations in the alcohol diet ranged from 3.5 to 4.06%, with a 0.14% increase every 2 weeks for a total of 8 weeks of feeding. Alcohol‐fed mice were fed ad libitum with an ethanol‐containing Lieber‐DeCarli liquid diet. The control mice in the PF group were fed an isocaloric control lipid diet with the amount consumed by the alcohol group on the previous day. On the day of sacrifice, the mice in the AF group received 5 g/kg BW ethanol gavage. Five hours later, the mice were sacrificed under anesthesia with pentobarbital solution (40 mg/kg BW) intraperitoneally.

### 4.14 Blood biochemistry

Liver function (ALT and AST) and lipid profile were measured as described previously,^30^ or by the National Resource Center for Non‐Human Primates at Kunming Institute of Zoology, Chinese Academy of Sciences as described previously.^59^

### 4.15 RNA‐sequencing and data analysis

Total RNA of each sample from the liver of mice treated with NAM and ABT263 was extracted using the TRIzol method. The polyA‐enriched RNA‐seq libraries were prepared for sequencing using the Illumina NovaSeq 6000 platform by Biolinker Technology (Kunming, China) and Majorbio Bio‐pharm Technology (Shanghai, China), respectively. The mouse genomic data mouse GRCm39 build and gene annotation information were downloaded from the Ensembl database (http://genome.ucsc.edu/). We used Trimmomatic to trim the adapters and obtained high‐quality clean reads for subsequent analysis. We mapped the clean reads onto the mouse genome Mus_musculus.GRCm39 build using the alignment software HISAT2 V2.2.1 (http://daehwankimlab.github.io/hisat2), and used samtools V1.6 (https://github.com/samtools) to convert sam files to binary bam files. Transcript was quantified from mapped data and gene expression level was calculated using featureCounts V2.0.6 software (https://subread.sourceforge.net/featureCounts.html). DEGs were identified using DESeq2 package (https://bioconductor.org/packages/release/bioc/html/DESeq2.html). A BH‐adjusted *p* value threshold of <0.01 was considered statistically significant. Gene ontology biological processes and pathways were analyzed using the Metascape database (https://metascape.org/). RNA‐seq data were publicly accessible from the National Genomics Data Center (NGDC) (GSE number: PRJCA031740 and PRJCA031825).

R (http://www.R‐project.org/) package ggplot2 was used to draw the differential gene volcano plot and the gene grouping comparison boxplot, and the Pheatmap package was used to draw the enrichment pathway and gene expression heatmap plot.

### 4.16 Statistical analysis

All statistical analyses were performed and figures were drawn using

GraphPad Prism v8. Data were presented as means ± standard error of the mean (SEM). Two‐tailed unpaired *t*‐test was used in comparisons between two experimental groups. The Welch test was used for Student's *t*‐tests that failed the normality test. One‐way analysis of variance (ANOVA) with Tukey's or Dunnett's post hoc test was used for comparisons between more than two groups. *p* < 0.05 was considered significant.

## AUTHOR CONTRIBUTIONS

N. M. G. and Q. C. D. performed most of the experiments. Y. Y., W. J. C., X. X. G., P. Y. Y., Y. Z. P., Q. N. L., X. Y. W., H. X., R. T., and M. T. Z. performed and analyzed some experiments. S. Y. P. analyzed the RNA‐sequencing data. N. M. G., Y. Y., M. N. O., and Y. H. H. wrote the manuscript. A. N., N. M. G., Q. C. D., and X. J. Z. revised the manuscript. Y. H. H. and S. T. L. conceived and supervised the study, and wrote and revised the manuscript. All authors have read and approved the final manuscript.

## CONFLICT OF INTEREST STATEMENT

The authors declare no conflicts of interest.

## ETHICS STATEMENT

The Institutional Animal Care and Use Committee of Zhejiang Chinese Medical University approved the animal experimental protocols, and the animals were maintained according to the guidelines of the Animal Experimental Center of Zhejiang Chinese Medical University (approval number 20220919–27).

## Supporting information



Supporting Information

Supporting Information

## Data Availability

RNA‐seq data were publicly accessible from the National Genomics Data Center (NGDC) (GSE number: PRJCA031740 and PRJCA031825). Other datasets used and analyzed during the current study are available from the corresponding author upon reasonable request.
