# Correction to “Therapeutic potential of nicotinamide and ABT263 inalcohol‐associated liver disease through targeting cellular senescence”

**DOI:** 10.1002/mco2.70969

**Published:** 2026-10-06

Gold NM, Ding Q, Yang Y, et al. Therapeutic potential of nicotinamide and ABT263 in alcohol‐associated liver disease through targeting cellular senescence. *MedComm*. 2025; 6: https://doi.org/10.1002/mco2.70086.

We originally ordered an antibody against CDKN2A (p16) and purchased ab51243 (Abcam). However, we subsequently discovered that this antibody specifically recognizes p16‐ARC (encoded by ARPC5), not CDKN2A. We therefore procured the correct CDKN2A antibodies from Proteintech (Cat. No. 10883‐1‐AP) and Santa Cruz Biotechnology (Cat. No. sc‐1661), selecting each based on species reactivity. All p16 Western blot data presented in the manuscript were generated using these two antibodies—not ab51243.

Regrettably, when preparing the Methods section, we inadvertently recorded ab51243 (Abcam) instead of the antibodies actually used. In Section 4.6 (“Western blotting”) of the “Materials and Methods,” the text reading “Primary antibody p16(ab51243) was purchased from Abcam (Cambridge, UK).” is incorrect and should be replaced with: “Primary p16 antibodies were purchased from Proteintech (10883‐1‐AP) and Santa Cruz Biotechnology (sc‐1661).”

We confirm that all experimental results reported in the paper are accurate; only the reagent information was recorded in error. This correction does not affect the data, interpretation, or conclusions of the article. The authors are grateful for the opportunity to correct this error.

We apologize for this error.

