# Supplementary material for: Therapeutic potential of nicotinamide and ABT263 in alcohol‐associated liver disease through targeting cellular senescence
Source: MedComm (2020). 2025 Feb 9;6(2):e70086. doi: 10.1002/mco2.70086 (PMC11808045; doi:10.1002/mco2.70086)
Supplement: Supplementary file 2 — Supporting Information [file MCO2-6-e70086-s001.docx]

**Therapeutic Potential of Nicotinamide and ABT263 in Alcohol-Associated Liver Disease through Targeting Cellular Senescence**

Naheemat Modupeola GOLD^1,2#^, Qinchao Ding^3,#^, Yang Yang^4,#^, Shaoyan Pu^1,5^, Wenjing Cao^3^, Xinxuan Ge^3^, Pengyun Yang^1,2^, Michael Ngozi Okeke^6^, Ayesha Nisar^1,2^, Yongzhang Pan^1,2^, Qiuni Luo^1^, Xiayan Wang^1,2^, Han Xu^1^, Rui Tian^7^, Meiting Zi^1^, Xingjie Zhang^8^, Songtao Li^3*^, Yonghan He^1,2*^

^1^Key Laboratory of Genetic Evolution & Animal Models, Key Laboratory of Healthy Aging Research of Yunnan Province, Kunming Institute of Zoology, Chinese Academy of Sciences, Kunming, Yunnan, China

^2^Kunming College of Life Science, University of Chinese Academy of Sciences, Kunming, China

^3^Department of Nutrition and Food Hygiene, School of Public Health， School of Public Health, Zhejiang Chinese Medical University, Hangzhou, Zhejiang, China

^4^Department of Biochemistry & Structural Biology, University of Texas Health Science Center, San Antonio, TX, USA

^5^Biodiversity Data Center of Kunming Institute of Zoology, Chinese Academy of Sciences, Kunming, Yunnan, China

^6^Guangdong Key Laboratory of Nanomedicine, Institute of Biomedicine and Biotechnology, Shenzhen Institute of Advanced Technology, Chinese Academy of Sciences, Shenzhen, China

^7^Department of Ultrasonography, The First Affiliated Hospital of Kunming Medical University, Kunming, Yunnan, China

^8^Key Laboratory of Medicinal Chemistry for Natural Resource, Ministry of Education, Yunnan Characteristic Plant Extraction Laboratory, Yunnan Key Laboratory of Research and Development for Natural Products, State Key Laboratory for Conservation and Utilization of Bio-Resources in Yunnan, School of Pharmacy and School of Chemical Science and Technology, Yunnan University, Kunming, Yunnan, China.

^#^These authors contributed equally to the work.

**Running title**: Therapeutic effect of NAM and ABT263 on ALD

***Corresponding author**:

Yonghan He, Kunming Institute of Zoology, Chinese Academy of Sciences, Kunming, Yunnan 650201, China

E-mail: [heyonghan@mail.kiz.ac.cn](mailto:heyonghan@mail.kiz.ac.cn)

Tel/Fax: +86-871-65118976

Or

Songtao Li, Department of Nutrition and Food Hygiene, School of Public Health, Zhejiang Chinese Medical University, Hangzhou, Zhejiang 310053, China

E-mail: [lisongtao@zcmu.edu.cn](mailto:lisongtao@zcmu.edu.cn)

Tel/Fax: +86-571-86633048

**Supplemental Figures**

**
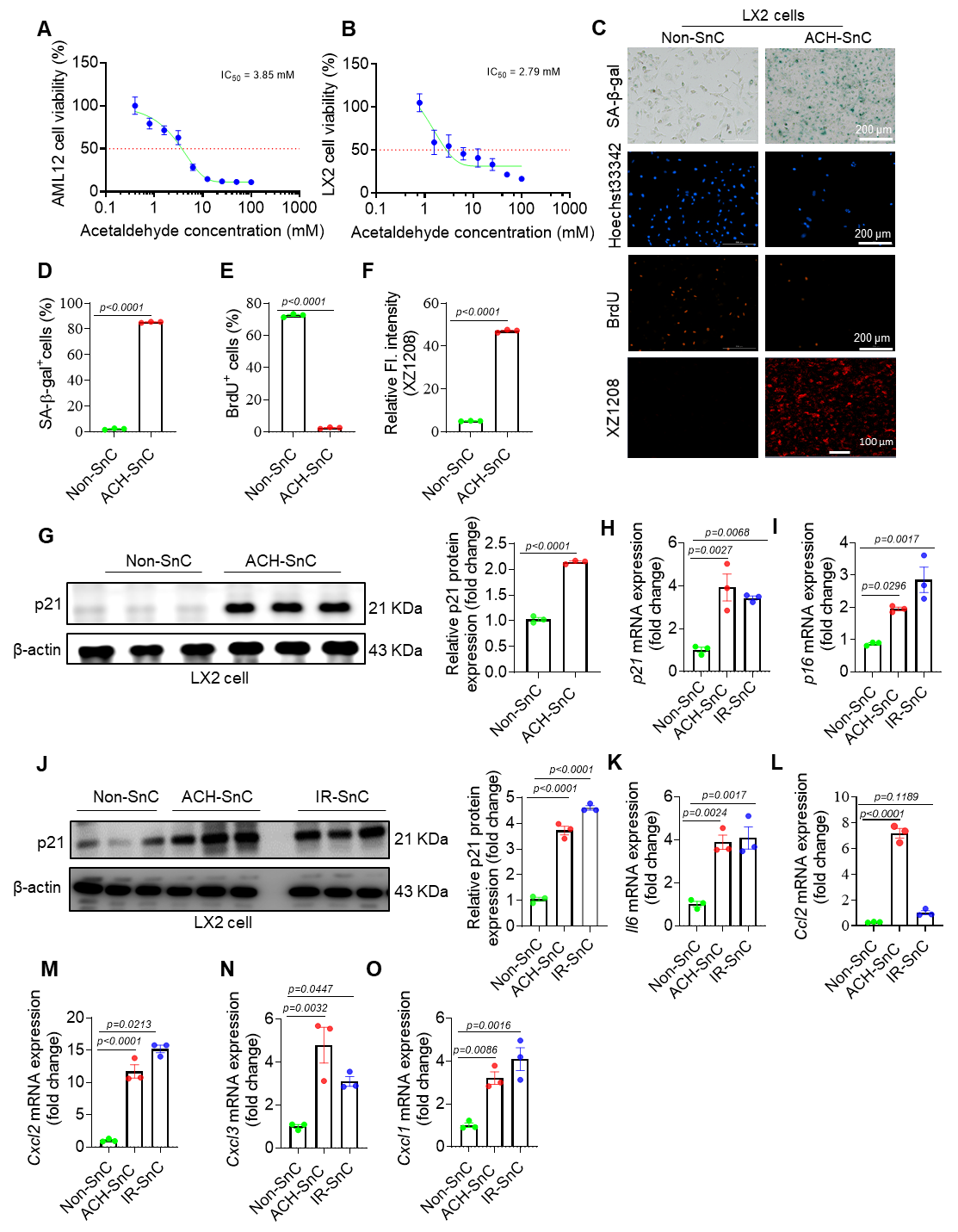
**

**Figure S1 Effect of** **acetaldehyde (ACH) on AML12 and LX2 cell viability and induction of senescence with ACH in LX2 cells.** (A-B) Cell viability assay showing the half-maximal inhibitory concentration (IC_50_) value of 24 h ACH treatment in AML12 (A) and LX2 (B) cells. (C) Representative images of SA-β-gal (scale bar = 200 μm), Hoechst33342 (scale bar = 200 μm), BrdU staining (scale bar = 200 μm), and XZ1208 labeling (scale bar = 100 μm) of non-senescent (Non-SnC) and acetaldehyde-induced senescent (ACH-SnC) LX2 cells. (D–F) Quantitative data analysis of SA-β-gal staining (D), BrdU staining (E), and relative fluorescent intensity (FI) of XZ1208 (F) in LX2 cells. Data are presented as mean ± SEM (n = 3 biologically independent samples) and analyzed with unpaired t-test or Welch's correction. (G) Western blotting assay and quantification of p21 expression in Non-SnC and ACH-SnC LX2 cells. (H-I) mRNA expression of *p21* (H) and *p16* (I) genes in Non-SnC, ACH-SnC and irradiated SnC (IR-SnC) LX2 cells. (J) Western blotting assay and quantification of p21 expression in Non-SnC, ACH-SnC, and IR-SnC LX2 cells. (K-O) Quantitative data analysis of mRNA expression of senescence-associated secretory phenotype (SASP) factors *Il6* (K), *Ccl2* (L), and *Cxcl2* (M), *Cxcl3* (N), and *Cxcl1* (O) in Non-SnC and ACH-SnC LX2 cells. Data are presented as mean ± SEM (n = 3 biologically independent samples) and analyzed with unpaired t-test or Welch's correction.

**
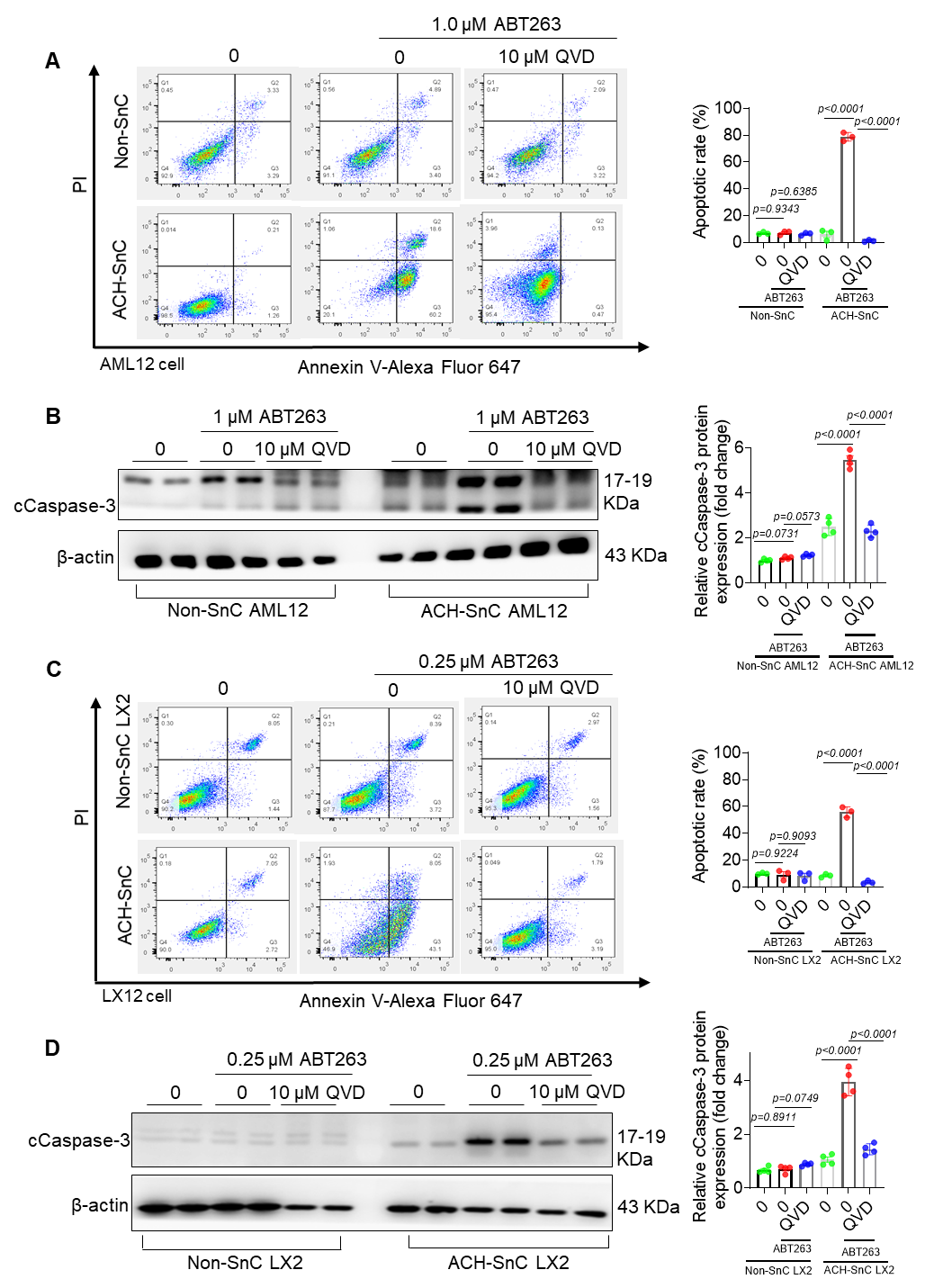
**

**Figure S2 ABT263 induces apoptosis in ACH-SnCs via activation of cleaved caspase-3.** (A-B) Flow cytometry and Western blotting assays of ABT263 induced apoptosis in ACH-SnC AML12 cells, which can be blocked by a pan-caspase inhibitor QVD. (C-D) Flow cytometry and Western blotting assays of ABT263 induced apoptosis in ACH-SnC LX2 cells, which can be blocked by a pan-caspase inhibitor QVD. Cells were pretreated with 10 µM QVD for 0.5 h, then treated with indicated concentrations of ABT263 for 24 h followed by Annexin-V and propidium iodide (PI) staining.


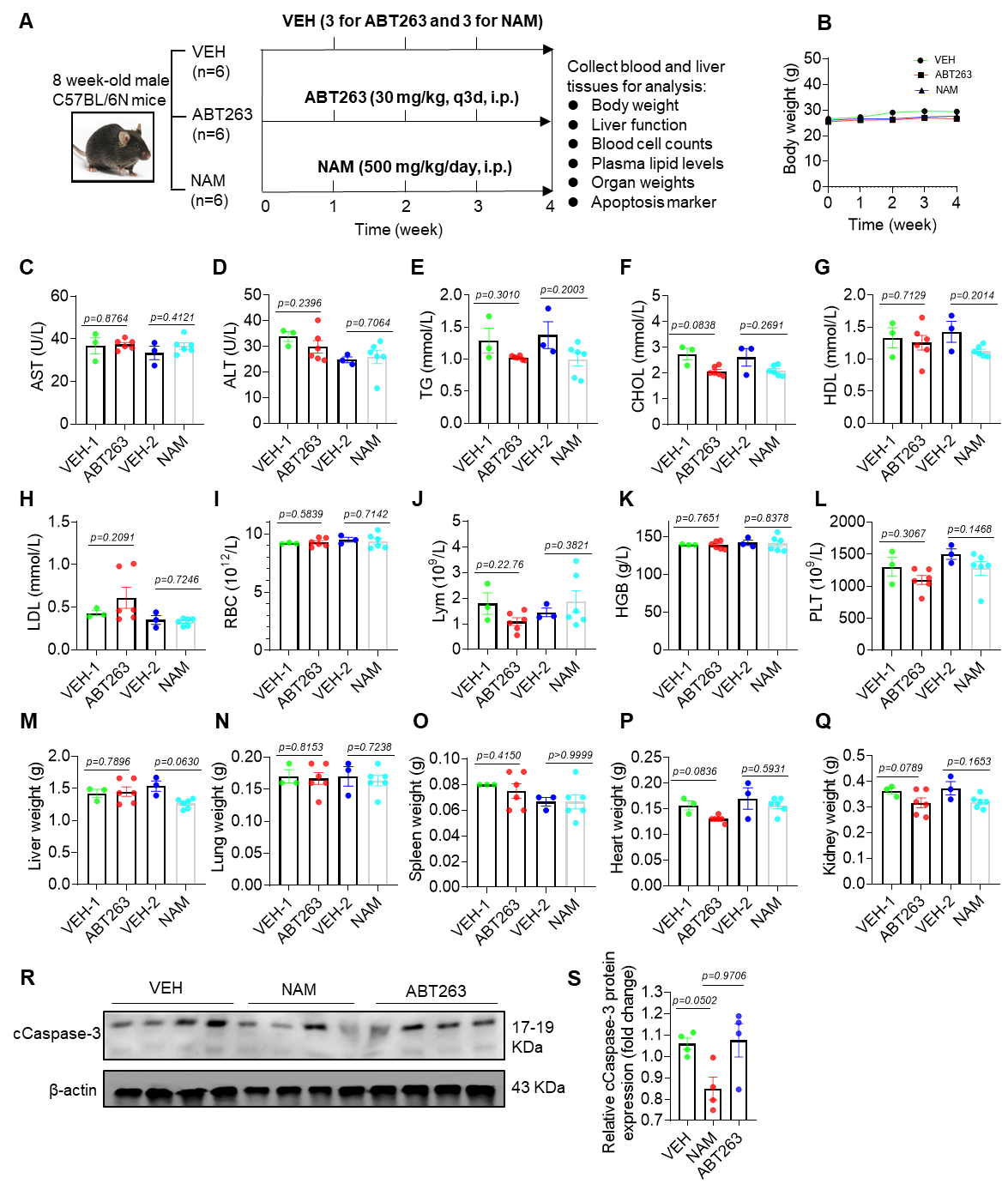


**Figure S3 Effect of ABT263 and nicotinamide (NAM) in normal mice.** (A) Experimental design: 8-week-old male mice were divided into three groups (n = 6 mice/group). They were given vehicle (VEH), ABT263 (30 mg/kg, i.p., q3d), and NAM (500 mg/kg/day, i.p.) for four weeks. Body weight, liver function, blood cell counts, lipids, organ weight, and apoptosis makers were assayed. (B) Body weight of mice across groups. (C–L) Analysis of liver function and blood parameters. Quantification of plasma levels of AST (C), ALT (D), TG (E), total cholesterol (CHOL) (F), HDL (G), LDL (H), red blood cells (RBCs) (I), lymphocytes (Lym) (J), hemoglobin (HGB)(K), and platelets (PLT) (L) across groups. (M–Q) Major organ weights across groups. (R-S) Effect of NAM and ABT263 treatments on the protein expression of cleaved caspase-3 (cCaspase-3).


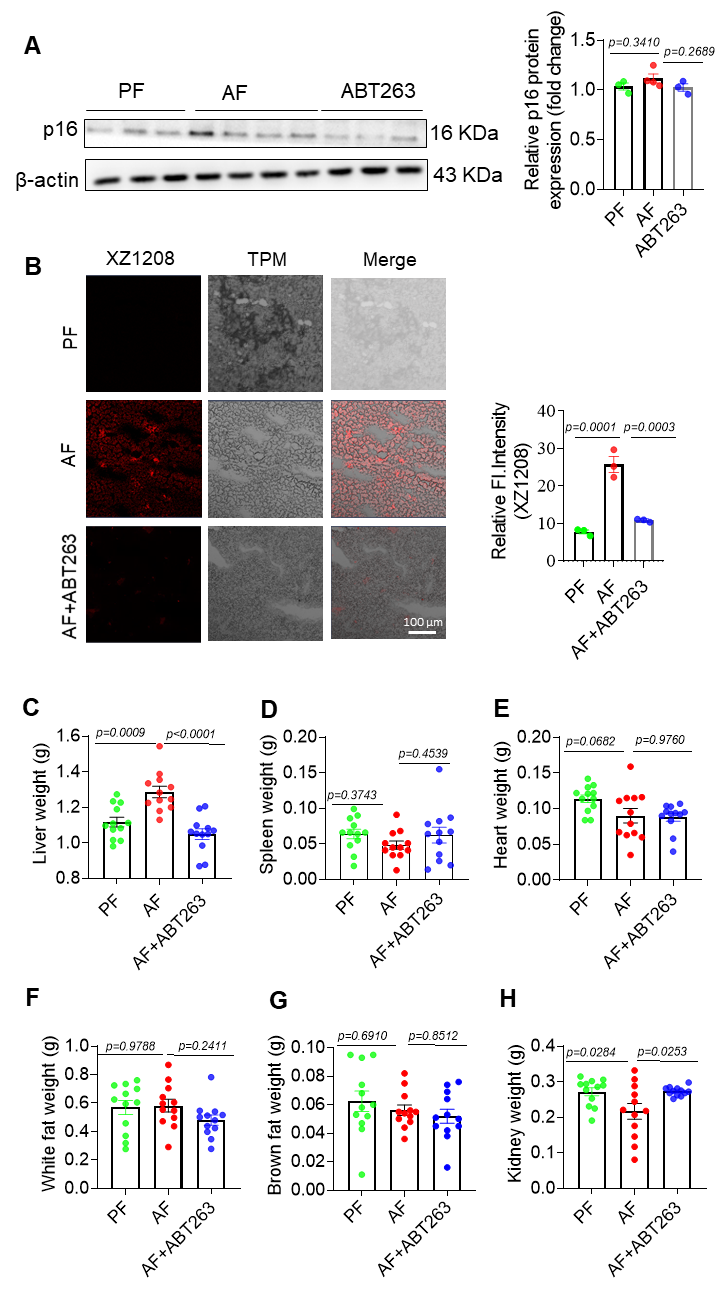


**Figure S4 Effect of ABT263 treatment on tissues and organs of ALD mice.** (A) Western blotting analysis of p16 expression in the liver of PF, AF, and ABT263 treated AF mice (n=3, 4 and 3 mice for PF, AF, and ABT263 treated groups, respectively). (B) XZ1208 staining of liver tissue of PF, AF, and ABT263 mice (n=3 mice for each group). (C-H) Organ and tissue weight in mice fed with alcohol and treated with ABT263. Data are presented as mean ± SEM (n = 12 mice/group) and were analyzed with one-way ANOVA using Dunnett’s multiple comparison test.


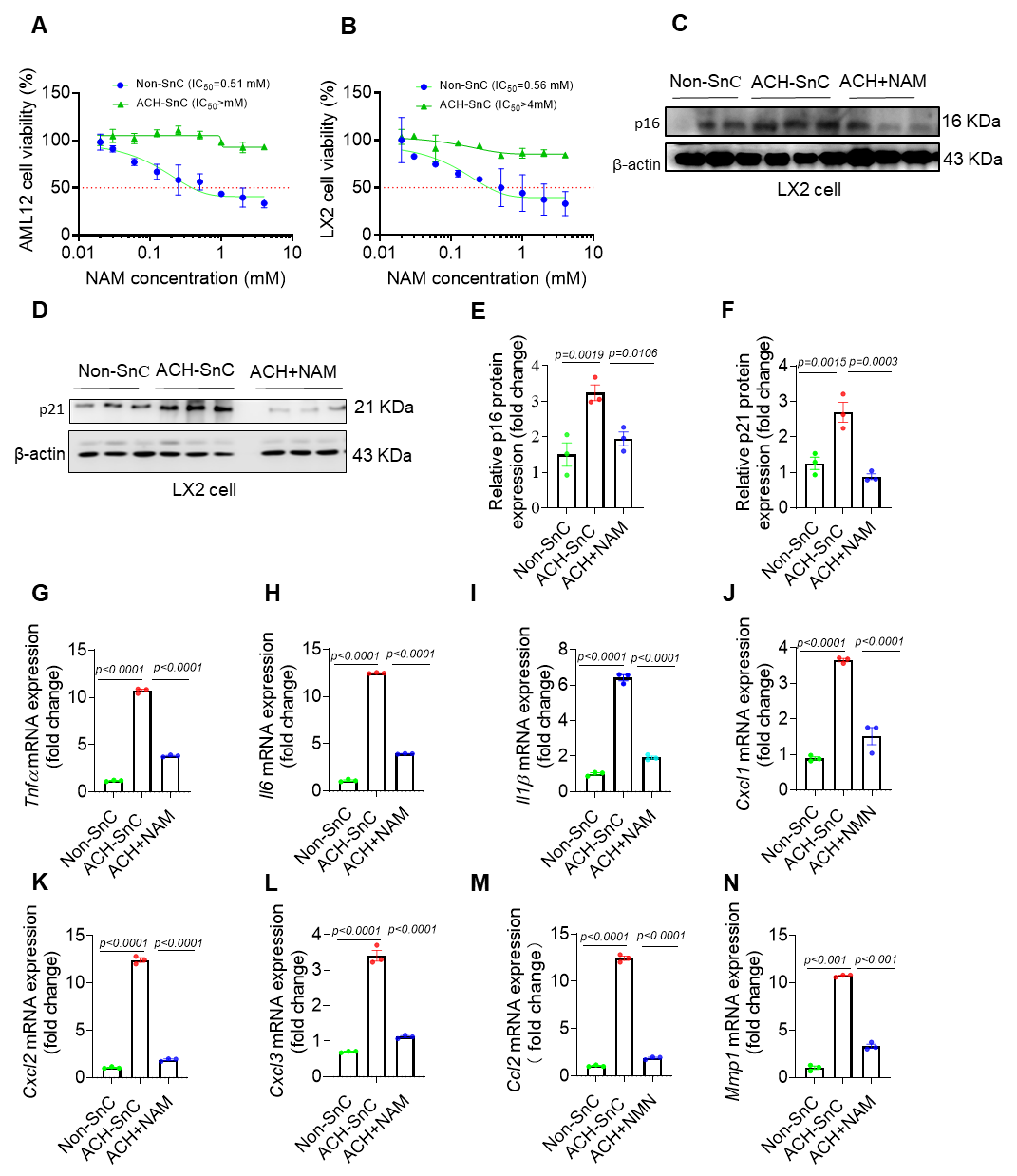


**Figure S5 NAM significantly suppresses senescence and senescence-associated secretory phenotype (SASP).** (A-B) Cell viability assay showing the half-maximal inhibitory concentration (IC_50_) value of 24 h treatment with indicated concentrations of NAM in Non-SnC and ACH-SnC AML12 (A) and LX2 (B) cells. (C–F) Western blotting assay of p16 (C) and p21 (D) and protein quantifications of p16 (E) and p21 (F) in Non-SnC and ACH-SnC LX2 cells treated with 0.5 mM NAM for 24 h. (G–N) Quantitative data of mRNA expressions of the SASP factors *Tnfα* (G), *Il6* (H), *Il1β* (I), *Cxcl1* (J), *Cxcl2* (K), *Cxcl3* (L), *Ccl2* (M), and *Mmp1* (N) in Non-SnC and ACH-SnC LX2 cells treated with NAM. Data are presented as mean ± SEM (n = 3 biologically independent samples), and analyzed with one-way ANOVA using Dunnett’s multiple comparison test.


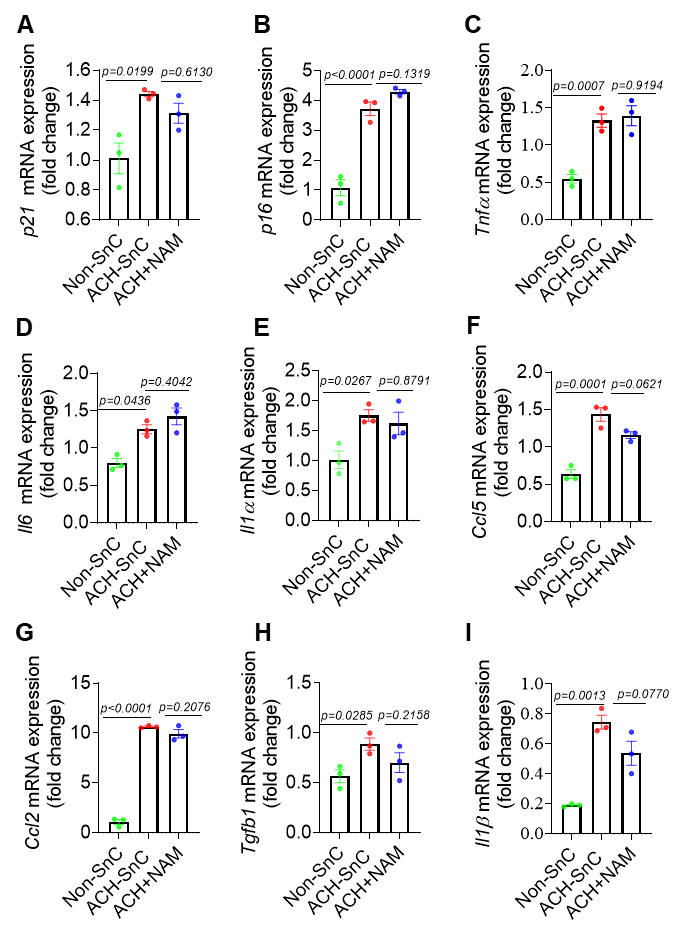


**Figure S6 Treatment with NAM after cells became senescent does not significantly suppress SASP in LX2 cells.** (A–I) Quantitative analysis of mRNA expressions of *p21* (A), *p16* (B), and the SASP factors *Tnfα* (C), *Il6* (D), *Il1α* (E), *Ccl5* (F), *Ccl2* (G), *Tgfb1* (H), and *Il1β* (I) in ACH-SnC LX2 cells. Cells were induced to be senescent with acetaldehyde (ACH), then treated with 0.5 mM NAM for 24 h. Data are presented as mean ± SEM (n = 3 biologically independent samples) and analyzed with one-way ANOVA using Dunnett’s multiple comparison test.


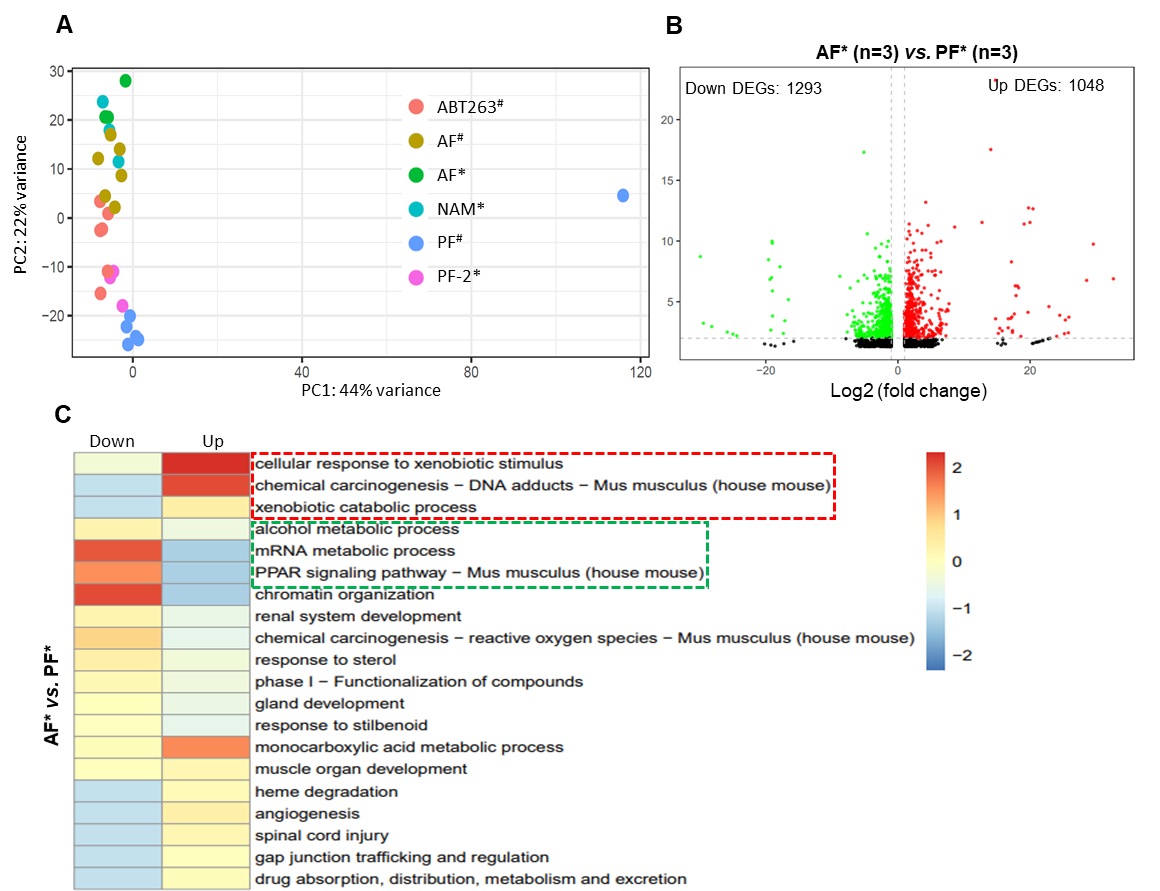


**Figure S7 Gene ontology (GO) analysis of differentially expressed genes (DEGs) in the alcohol-fed (AF) and paired-fed (PF) mice groups.** (A) Principal components analysis (PCA) on samples. ^#^ and * indicates samples belonging to the same batch, respectively. One outlier of the PF^#^ samples was removed from the analysis. (B) Volcano plot showing differentially expressed genes (DEGs) in the liver of AF and PF mice. Red dots represent down DEGs, green represent up DEGs, and black represent non-DEGs. (C) Enrichment of DEGs in the liver of AF versus PF mice. Red and green dashed lines indicate up- and down-regulated enrichment of DEGs, respectively. The legend on the right represents the value range after the data were normalized by the scale function within the Pheatmap package. The color varies from blue to red, signifying that the differences are becoming gradually significant.

**
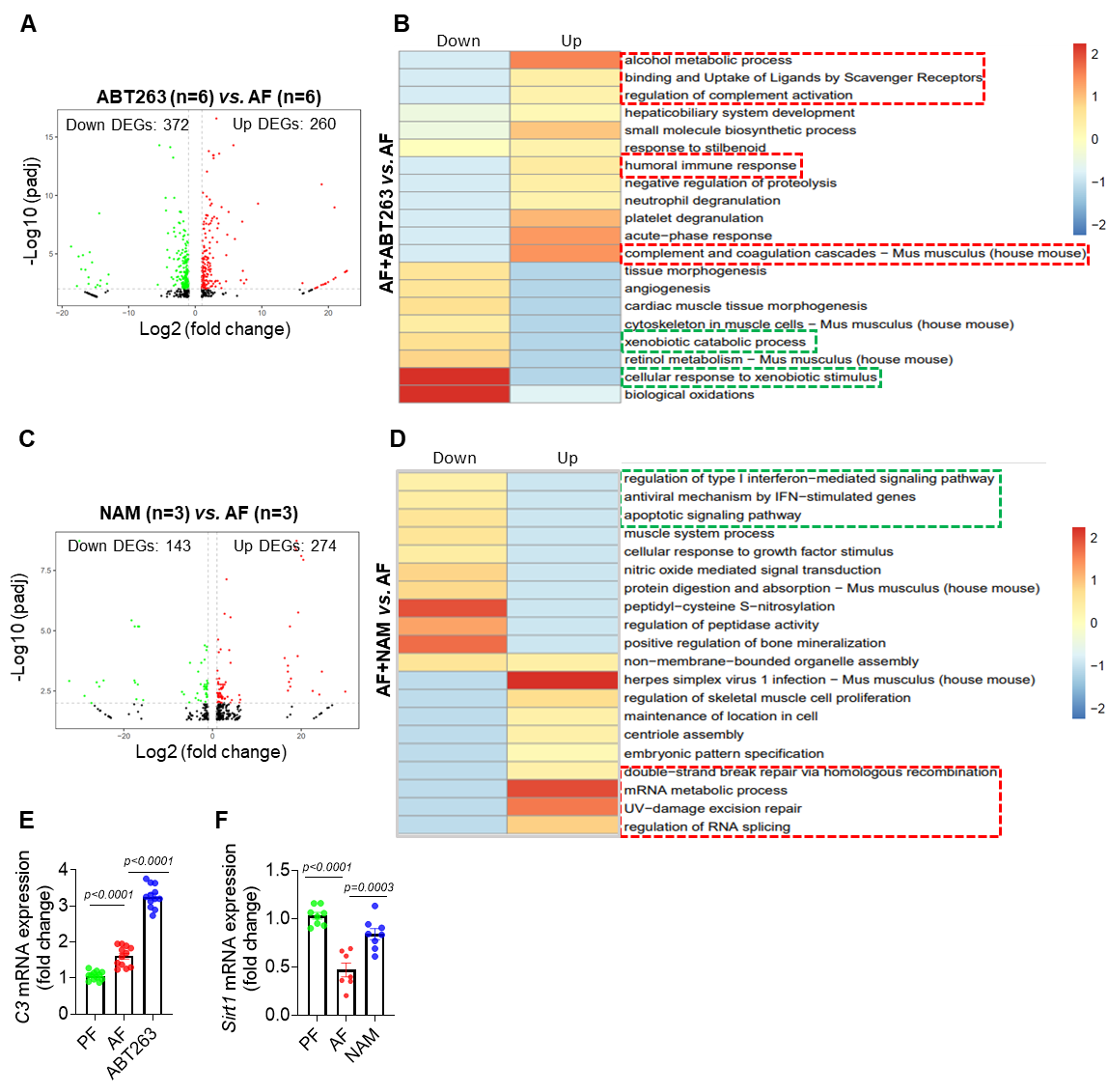
Figure S8 Gene ontology (GO) analysis of the differentially expressed genes (DEGs) in the alcohol-fed (AF) and paired-fed (PF) mice groups.** (A-B) Differentially expressed genes (DEGs) and their enrichments in the liver of AF and ABT263 treated mice. (C-D) Differentially expressed genes (DEGs) and their enrichments in the liver of AF and nicotinamide (NAM) treated mice. Red dots represent up DEGs, green represent down DEGs, and black represent non-DEGs. (E-F) qPCR validation of mRNA expression of *C3* and *Sirt1* in the liver of PF (n = 8 mice/group), AF (n = 7 mice/group), and ABT263- (n = 12 mice/group) or NAM-treated mice (n = 8 mice/group. Data are presented as mean ± SEM and analyzed with one-way ANOVA using Dunnett’s multiple comparison test. The legend on the right represents the value range after the data were normalized by the scale function within the Pheatmap package. The color varies from blue to red, signifying that the differences are becoming gradually significant.

**
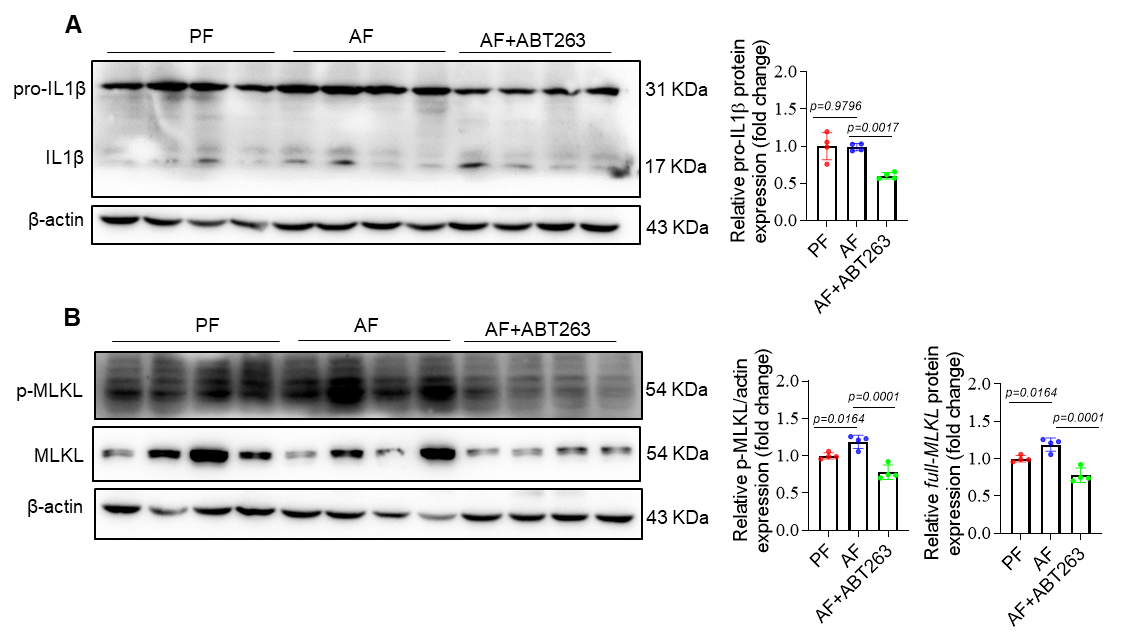
**

**Figure S9 Effect of ABT263 and NAM on the activation of IL1β and MLKL.** (A-B) Western blotting assay and quantification of the relative expression of pro-IL1β (A), p-MLKL and full MLKL expression (B) in the liver of alcohol-fed (AF), paired-fed (PF), and ABT263-treated mice (n = 4 for PF, AF and ABT263 treated groups, respectively). Data are presented as mean ± SEM and analyzed with one-way ANOVA using Dunnett’s multiple comparison test.
